# Interventions addressing men, masculinities and gender equality in sexual and reproductive health and rights: an evidence and gap map and systematic review of reviews

**DOI:** 10.1136/bmjgh-2019-001634

**Published:** 2019-09-11

**Authors:** Eimear Ruane-McAteer, Avni Amin, Jennifer Hanratty, Fiona Lynn, Kyrsten Corbijn van Willenswaard, Esther Reid, Rajat Khosla, Maria Lohan

**Affiliations:** 1 School of Nursing and Midwifery, Queen's University Belfast, Belfast, United Kingdom; 2 Centre for Evidence and Social Innovation (CESI), Queen's University Belfast, Belfast, United Kingdom; 3 Department of Reproductive Health and Research (RHR), World Health Organization, Geneve, Switzerland

**Keywords:** Public Health, Systematic review

## Abstract

**Objectives:**

Working with men/boys, in addition to women/girls, through gender-transformative programming that challenges gender inequalities is recognised as important for improving sexual and reproductive health and rights (SRHR) for all. The aim of this paper was to generate an interactive evidence and gap map (EGM) of the total review evidence on interventions engaging men/boys across the full range of WHO SRHR outcomes and report a systematic review of the quantity, quality and effect of gender-transformative interventions with men/boys to improve SRHR for all.

**Methods:**

For this EGM and systematic review, academic and non-academic databases (CINAHL, Medline, PsycINFO, Social Science Citation Index-expanded, Cochrane Library, Campbell Collaboration, Embase, Global Health Library and Scopus) were searched using terms related to SRHR, males/masculinities, systematic reviews and trials (January 2007–July 2018) with no language restrictions for review articles of SRHR interventions engaging men/boys. Data were extracted from included reviews, and AMSTAR2 was used to assess quality. Outcomes were based on WHO reproductive health strategy.

**Results:**

From the 3658 non-duplicate records screened, the total systematic reviews of interventions engaging men/boys in SRHR was mapped through an EGM (n=462 reviews) showing that such interventions were relatively evenly spread across low-income (24.5%), middle-income (37.8%) and high-income countries (37.8%). The proportion of reviews that included gender-transformative interventions engaging men/boys was low (8.4%, 39/462), the majority was in relation to violence against women/girls (n=18/39, 46.2%) and conducted in lower and middle-income countries (n=25/39, 64%). Reviews of gender-transformative interventions were generally low/critically low quality (n=34/39, 97.1%), and findings inconclusive (n=23/39, 59%), but 38.5% (n=15/39) found positive results.

**Conclusion:**

Research and programming must be strengthened in engagement of men/boys; it should be intentional in promoting a gender-transformative approach, explicit in the intervention logic models, with more robust experimental designs and measures, and supported with qualitative evaluations.

**Key questions**

**What is already known?**

- Engagement of men/boys alongside women in gender-transformative programming is fundamental to addressing gender inequality and sexual and reproductive health and rights (SRHR) for all.

**What are the new findings?**

- The paper offers the first interactive evidence and gap map of the total systematic review evidence on interventions engaging men/boys mapped across the full range of WHO SRHR outcomes.
- A minority of reviews included gender-transformative interventions with men/boys (8.4%, 39/462 reviews), of which 39% reported positive results, but the majority was mixed or inconclusive, and the overall reporting quality of reviews was low.
- Review evidence engaging men/boys is approximately equally prevalent in low-income, middle-income and high-income countries, but gender-transformative approaches with men/boys is particularly likely to be found in low-income and middle-income countries.

**What do the new findings imply?**

- Future research and programming with men/boys needs to promote a gender-transformative approach, explicit in the intervention logic models, with more robust experimental designs and measures, supported with qualitative evaluations.
- Greater partnership is required between programme implementers and researchers in order to realise the potential for engaging men/boys in promoting gender equality to improve SRHR for all.

## Introduction

The case for addressing gender equality as part of a human rights-based approach to improving health, including sexual and reproductive health (SRH), has been a long-standing guiding principle in the feminist literature on gender and development and significantly foregrounded in global public health since before the 1994 International Conference on Population and Development (ICPD) in Cairo.^1–9^ The conference marked a paradigm shift in global health away from an overarching concern with population control in low-resource countries to a human rights-based approach aimed at empowering women to control their fertility and their access to safe childbearing, while making explicit too the need to engage men to make this a reality.

Since then, too, the focus on addressing gender inequality in health programming has become more clearly conceptualised as a gender-transformative approach. The concept of gender-transformative approaches was first coined by Dr Geeta Rao Gupta^10^ in the context of the HIV/AIDS epidemic and has since gained traction in international health and development policy.^6 11 12^ The WHO defines a gender-transformative approach as one ‘that address the causes of gender-based health inequities through approaches that challenge and redress harmful and unequal gender norms, roles, and power relations that privilege men over women’.^11^ Men are also implicated in the harmful consequences of gender inequality, harming their own and other men’s health and the health of their female partners as a result of narrow and constraining definitions of what it means to be a man, therefore gender-transformative approaches also benefit men in broadening the interpretation of masculinity and the socially acceptable ways in which masculinity can be expressed.^13 14^

Just as in the original definition offered by Rao Gupta, the WHO definition of a gender-transformative approach is derived from considering a continuum of approaches to addressing gender equality in health programming. In the WHO^15^ definition, these are: a *gender unequal* approach that perpetuates gender inequality by reinforcing unbalanced norms, roles and relations; a *gender-blind* approach that ignores gender norms, roles and relations and thereby often reinforces gender-based discrimination; a *gender-sensitive* approach that considers gender norms, roles and relations but does not address inequality generated by unequal norms, roles or relations; a *gender-specific approach* that considers women’s and men’s specific needs or roles but does not seek to change these roles; and a *gender-transformative approach* that considers gender norms, roles and relations for women and men, as does gender-specific and gender-sensitive, but is distinguished by the imperative to challenge gender inequality. A gender-transformative approach seeks to challenge gender inequality by transforming harmful gender norms, roles and relations through inclusion in programming of strategies to foster progressive changes in power relationships between women and men.

The underpinning rationale of addressing gender inequality is because it is a key determinant of the health of men and women of all gender identities and sexualities yet generally disproportionately disadvantages the opportunities and outcomes for women and girls, including in the particular field of sexual and reproductive health and rights (SRHR).^16–22^ However, a gender-transformative approach also prompts an explicit focus on the roles of men/boys in transforming gender inequality to improve men’s health and especially SRHR. There is increasing recognition that men and boys can play a role as either supporting and championing or damaging and denying the health and rights of women and girls. Hence, focusing on boys/men through a gender-transformative approach goes beyond a men’s health focus or the inclusion of men as partners of women with respect to SRH decision making.^23–31^ Despite more than a decade of gender-transformative programming on engaging men/boys in several areas of health including SRHR, there is a paucity of evidence on the effectiveness of interventions to improve SRHR outcomes; how best to engage men/boys from a gender-transformative perspective; and what works and for which SRHR health outcomes. Therefore, when considering how best to promote SRHR globally through gender-transformative programming, it is important to take stock of the evidence and identify policy, programme and research implications.

The aim of this paper is to first generate an interactive evidence and gap map (EGM) of the total systematic review evidence of interventions engaging men/boys mapped across the full range of WHO SRHR outcomes and to identify those reviews that contain gender-transformative interventions relating to each SRHR outcome. This leads to the second aim of this paper, which is to report a systematic review of the quantity, quality and effect of gender-transformative interventions with men/boys to improve SRHR. Our exclusion of programmes/interventions that we considered not to be gender-transformative does not mean such interventions are not of value or have not shown promise. The choice to focus on identifying gender-transformative interventions, and not the entire WHO continuum from gender unequal to gender-specific, was, however, informed by global policy interest in addressing gender inequality in health programming.

We addressed the following questions:

1. What is the state of the evidence on interventions designed to engage men/boys across all WHO SRHR outcomes?
2. What is the proportion of these interventions that are explicitly gender-transformative?
3. What is the methodological quality of gender-transformative interventions with men/boys?
4. To what extent are gender-transformative interventions with men/boys effective in positively impacting SRHR outcomes?

## Methods

The decision to conduct a review of reviews rather than of primary intervention studies was guided by the necessity of including the broad scope of all seven WHO defined SRHR outcomes (listed below). For the EGM, we searched CINAHL, Medline, PsycINFO, Social Science Citation Index-expanded, Cochrane Library, Campbell Collaboration, Embase, Global Health Library, Scopus and Google Scholar for systematic reviews (1 January 2007–31 July 2018). There were no language restrictions. Bibliographies of included reviews were screened. The search dates were based on a previous WHO review of the evidence.^32^ Review papers deemed eligible for inclusion were systematic reviews synthesising findings from experimental studies (randomised controlled trials (RCTs)/quasi-experimental) that included men/boys and assessed the effect on SRHR outcomes. The decision to include reviews including RCTs and quasi-experimental studies only was based on the need to evaluate high-quality evidence on intervention effectiveness. If a review included additional non-experimental studies, data were only extracted for experimental studies. A review was considered systematic when it contained a systematic search, characterised by the reporting of a predetermined search strategy, specifying the location of the search and stating the numbers and reasons for excluding papers from the final synthesis (eg, Preferred Reporting Items for Systematic Reviews and Meta-Analyses (PRISMA) flow chart). The population of interest included males of all ages, irrespective of sexual orientation. Comparators included either no interventions, services as usual or alternative services.

The eligibility criteria for the systematic review of gender-transformative interventions was as above, but limited to the subset of reviews that included interventions using a gender-transformative approach, that is, included evaluations of interventions that included ways to transform harmful gender norms, gender practices, gender inequality and/or addressed the causes of gender-based inequities within the interventions.^11^ Where reviews did not exclusively focus on gender-transformative interventions, data were extracted for relevant gender-transformative interventions only.

We operationalised the WHO^15^ definition of gender-transformative programming:

- Considers gender norms, roles and relations for women and men and that these affect access to and control over resources.
- Considers women’s and men’s specific needs.
- Addresses the causes of gender-based health inequities.
- Includes ways to transform harmful gender norms, roles and relations.
- The objective is often to promote gender equality.
- Includes strategies to foster progressive changes in power relationships between women and men.

Each review and any additional data tables and appendices were read by two authors independently to identify elements of interventions that were articulated as transforming gender norms, masculinity norms and/or unequal power relations between women and men. Hence, to the extent possible in an exercise of this nature relying on review level descriptions of interventions in a peer-reviewed article, we extracted gender-transformative interventions as per the definition provided by WHO.^15^ Current reviewers did not rely on included review authors’ classifications of a review being gender-transformative or not. online supplementary appendix 1 includes a list of included reviews categorised as gender-transformative; this list is subcategorised based on the intent of the review, that is, category A: reviews that explicitly sought to include gender-transformative interventions and category B: reviews that did not explicitly seek to identify gender-transformative interventions for their reviews, yet at least one gender-transformative intervention was included due to a focus in the review on an outcome, such as HIV or domestic violence, where evaluations of gender-transformative interventions have been conducted.

10.1136/bmjgh-2019-001634.supp1 Supplementary data

The SRHR outcomes of interest were based on the WHO Reproductive Health Strategy^33^:

1. Helping people realise their desired family size (contraception and family planning; prevention and treatment of infertility).
2. Ensuring the health of pregnant women/girls and their new-born infants (maternal and infant mortality; preventing complications in pregnancy, childbirth and postnatal period).
3. Preventing unsafe abortion.
4. Promoting sexual health and well-being (prevention of reproductive tract and sexually transmitted infections; HIV/AIDS; and interventions promoting sexual well-being, for example, treatments for erectile dysfunction. Excluding conditions not acquired sexually, for example, testicular and prostate cancers and more general men’s health conditions)
5. Promoting SRH in disease outbreaks (prevention of sexual transmission of Zika and Ebola viruses).
6. Promoting healthy adolescence for a healthy future (covering all SRHR outcomes with a specific focus on adolescents).
7. Preventing and responding to violence against women/girls (intimate partner violence (IPV); domestic violence and sexual coercion/violence) and harmful practices (ie, female genital mutilation; child, early and forced marriage; and IPV in males).

Search terms related to SRHR were adapted from a previous systematic review of SRH interventions in humanitarian crises conducted by Warren and colleagues.^34^ Terms related to males and masculinities were developed and tested in a number of databases. An edited Pearl Harvesting string was used to identify systematic review papers.^35^ Search terms are reported in online supplementary appendix 2. This review title was registered,^36^ and protocol was published^37^ with the Campbell Collaboration.

### Data analysis

Four authors (ER-M, ML, KCvW and ER) and one trained researcher (Dr Conall O’Rourke, see Acknowledgements) applied the inclusion and exclusion criteria when screening titles, abstracts and full-text results for eligibility using Distiller Systematic Review Software (2017). One author arbitrated disagreement. Inter-rater reliability score was considered acceptable; at full-text screening, the weighted overall kappa score was 0.60 (original kappa) and 0.97 after moderation.

Double-blind data extraction was conducted by two authors (ER-M and KCvW). The outcomes of interest in the EGM were: WHO outcome domains; types of studies included (RCTs only or mixed designs); study resource setting (high/middle/low-income countries, as per World Bank categorisations,^38^ and whether the approach in the review identified gender-transformative interventions or not (online supplementary appendix 3)). However, in nine of the included reviews, insufficient intervention detail was available at review level to extract individual intervention-level data.

For the reviews engaging men/boys through a gender-transformative approach, we extracted the above information along with key components and theoretical rationale of interventions included, settings and participants, key findings and recommendations of the reviews (online supplementary appendix 4).

The AMSTAR2 tool^39^ was used to assess the methodological quality of the subset of reviews including interventions that used a gender-transformative approach with men. Double-blind quality assessment was conducted by two reviewers (KCvW and FL) with an inter-rater reliability of 83.2% achieved for individual items, with full agreement in the overall rating of quality for each included systematic review. Any differences in the appraisal of individual items were discussed until agreement was reached.

### Role of the funding source

The review was funded through a grant from the WHO Human Reproduction Programme (HRP) that specialises in SRHR research. Staff from HRP specialising in gender equality and human rights provided technical oversight on the study design, data analysis and data interpretation. The corresponding author had full access to all the data in the study and had final responsibility for the decision to submit for publication. Findings were presented to the WHO HRP Gender and Rights Advisory Panel (GAP) in January 2019, composed of international experts in gender equality and human rights in SRHR. The GAP provided further inputs on the findings of the review.

## Results

**Figure 1:**
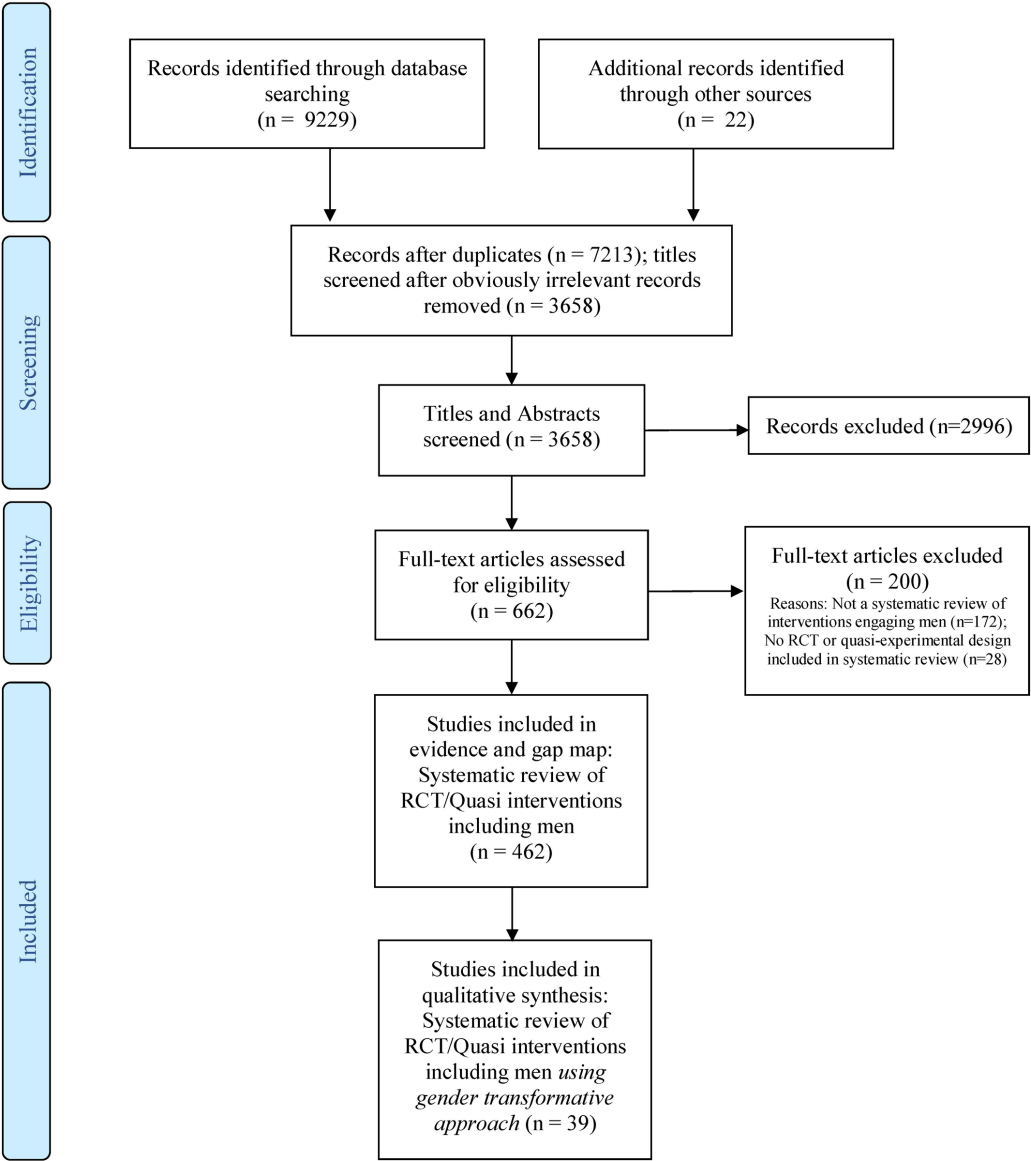
PRISMA flow diagram. RCTs, randomised controlled trials.

On screening of 3658 non-duplicate records and full-text screening of 662 full texts, 462 eligible reviews on engaging men/boys in SRHR were included in the EGM. Thirty-nine of the systematic reviews reported on gender-transformative interventions that engaged men/boys and were included in the systematic review of reviews. Figure 1 illustrates the PRISMA flow chart documenting search, screening and reasons for exclusion.

Findings responding to the first question (ie, the state of the evidence on interventions designed to engage men/boys across all SRHR outcomes) are presented as an EGM. The EGM, with a visual interactive summary of all systematic reviews involving men/boys to improve SRHR, categorised under the identified seven WHO SRHR outcome domains, can be accessed here: http://srhr.org/masculinities/rhoutcomes/. This EGM also identifies the reviews that contain gender-transformative interventions. A second EGM further categorising the same data by resource settings (high-income countries (HICs); middle-income countries (MICs); and low-income countries (LICs)) is provided in http://srhr.org/masculinities/wbincome/.

The EGM results demonstrate that the overall review evidence on engaging men/boys in improving SRHR had a relatively balanced spread across economic context. Over one-third of the total review evidence on engaging boys/men in SRHR is in HICs (n=242 reviews), over one-third in MICs (n=242 reviews) and approximately one quarter in LICs (n=157 reviews). However, the EGM results demonstrate that the evidence on engagement of men/boys varies considerably across the SRHR outcomes. The WHO SRHR outcomes with the greatest quantity of review evidence on engaging men/boys are: promoting sexual health and well-being (68.2% of reviews), followed by those measuring outcomes related to desired family size (31.4%) and healthy adolescence (25.1%). Fewer reviews covered outcomes related to violence against women/girls (14.5%) and health of pregnant women and children (9.1%). Only two reviews were found that looked at outcomes related to preventing unsafe abortion and no reviews looked at SRH in disease outbreaks (table 1).

**Table 1:** Reviews on engaging men in relation to WHO sexual and reproductive health and rights outcomes and the proportion of which is gender-transformative

WHO SRHR outcome	Number and percentage of outcomes observed across all reviews (n=462)*	Ratio of gender transformative to non-gender transformative
Promoting sexual health and well-being	315 (68.2%)	1:16.5
Desired family size	145 (31.4%)	1:12.2
Healthy adolescence	116 (25.1%)	1:5.8
Health of pregnant women, infants and girls	42 (9.1%)	1:5.0
Violence against women/girls	67 (14.5%)	1:1.2
Preventing unsafe abortion	2 (0.4%)	–
Sexual and reproductive health in disease outbreaks (ie, Ebola and Zika)	0 (0)	–

*Reviews could cover multiple domains. Percentages of reviews in outcome domains were calculated as a percentage of the total number of reviews (n=462), for example, 315 of 462 reviews (68.2%) contained interventions on promoting sexual health and well-being.

SRHR, sexual and reproductive health and rights.

Turning to the second question relating to the evidence for gender-transformative approaches with men, a very small proportion of reviews engaging men/boys contained gender-transformative interventions. Of the 462 reviews, only 39 (8.4% total reviews) included an intervention engaging men/boys in SRHR adopting a gender-transformative approach (online supplementary appendix 5). The greatest imbalance in reviews including gender-transformative interventions engaging men/boys (as measured by the ratio of gender-transformative to non-gender-transformative reviews in each WHO SRHR domain) is for outcomes related to promoting sexual health, desired family size and healthy adolescence (table 1). Of significance, however, reviews of interventions addressing violence against women/girls (VAWG) are almost equally likely to be gender transformative than non-gender transformative. Also, the gender-transformative review evidence engaging men/boys in SRHR was more likely to be from low-income and middle-income countries (LMICs) with fewer from HICs. Only 14 of the 39 reviews (35.9%) contained studies from HICs.

Focusing on the 39 reviews of gender-transformative interventions engaging men/boys, we summarise their characteristics in terms of their aims, number of gender-transformative interventions included, study designs and outcomes, methodological quality (AMSTAR2 score) and conclusions about effectiveness (table 2). The majority of reviews including gender-transformative interventions were rated low or critically low quality (n=34), largely due to inadequate reporting of methodological details. The agreed ratings for each item of AMSTAR2, as well as the overall rating of included reviews can be found in online supplementary appendix 6. As the checklist was designed to assess the methodological quality of systematic reviews, it was not applicable for assessing four papers identified as reviews of reviews.^40–43^

**Table 2:** Characteristics of included reviews

Review	Aim	Date range of search	No. of included studies (RCT, n; quasiexperimental, n; other, n)	No. of included studies=gender-transformative for men+RCT/quasiexperimental	WHO domain (1–7)*	AMSTAR2 summary score	Conclusions
Arias *et al*^53^	‘To perform a meta-analysis to learn the state-of-the-art of the efficacy of batterer treatment programmes from the 1975 to 2013 by assessing studies measuring treatment efficacy in terms of the recidivism rate’.^53^	1975–2013	19 (6; 13; 0)	15	7	Critically low	Inconclusive/ mixed
Anderson *et al*^44^	‘The aim of this paper is to critically examine interventions that have been studied in sub-Saharan Africa to address both HIV and IPV with the purpose of identifying interventions that might be implemented by nurses in Africa and other settings’.^44^	2001–2012	17 (NR; NR; NR)	Unclear	4, 7	Critically low	Positive effect
Arango *et al*^43^	‘To synthesise and distil that information in order to provide readers with a more unified and thorough understanding of the evidence on various interventions for preventing and reducing VAWG’.^43^	2000–2013	58: 23 systematic reviews, 35 comprehensive reviews (other study designs, 192)	Unclear	6, 7	N/A	Inconclusive/ mixed
Bacchus *et al*^54^	‘To identify interventions that have measured outcomes for both IPV and CM and programme components that may have contributed to positive outcomes. Due to the fact that current evidence focuses largely on high-income countries, this paper focuses on interventions in LMIC to build the knowledge base in less developed settings’.^54^	2010–2015	9 (5; 0; 4)	3	7	Critically low	Positive effect
Bakrania *et al*^40^	‘This EGM collates the evidence base for adolescent interventions in LMICs where research is particularly scarce. The thematic scope broadly corresponds with the UNICEF adolescent well-being outcome domains of protection, participation and livelihoods (excluding transferable skills and youth employment-related interventions and outcomes as other EGMs address these). Outcomes relating to the enabling environment for adolescents are also included to capture the contextual influences that might affect the wellbeing of adolescents’.^40^	2000–(end date NR)	71 impact evaluations, 3 systematic reviews (45; 26; three systematic reviews)	Unclear	6, 7	N/A	Inconclusive/ mixed
Bourey *et al*^55^	‘This systematic review aims to synthesise peer-reviewed evidence on the quantitative impact of structural interventions to prevent male-perpetrated IPV against women in LMIC’.^55^	2000–2015	20 (13; 2; 5)	15	6, 7	Critically low	Positive effect
Casey *et al*^56^	‘The overarching purpose of this review is to apply a gender-transformative lens to summarising current literature regarding effective strategies for promoting men’s anti-GBV engagement’.^56^	NR	10 (NR; NR; NR)	10	6, 7	Critically low	Positive effect
Chatterjee^57^	‘Objective of this review was to systematically review and synthesise the evidence of what could work to prevent child marriages in India’.^57^	NR	15 (6; 9; 0)	Unclear	1, 6, 7	Critically low	Inconclusive/ mixed
DeGue *et al*^58^	‘The goal of this review is to identify and summarise the best available evidence on specific sexual violence primary prevention strategies’.^58^	1985–2012	140 (82; 35; 23)	Unclear	6, 7	Critically low	Inconclusive/ mixed
Denison *et al*^59^	‘What is the effectiveness of interventions designed to reduce the prevalence of female genital mutilation/cutting compared to no or any other intervention?’^59^	NR	9 Publications (6 studies) (NR; NR; NR)	4	7	Low	Inconclusive/ mixed
Dworkin *et al*^24^	‘We seek to evaluate gender-transformative interventions as they impact four sets of outcomes: HIV/STI outcomes, violence perpetration, sexual risk behavior, and psychosocial markers of gender equity (norms and attitudes)’.^24^	NR	15 (3; 5; 7)	8	4, 6, 7	Critically low	Positive effect
Ellsberg *et al*^60^	‘We review evidence for interventions to reduce the prevalence and incidence of violence against women and girls’.^60^	NR	Unclear – 22 stated in text but only 14 included in table (9; 5; 0)	9	6, 7	Critically low	Positive effect
Feder *et al*^50^	‘To assess the effects of post-arrest court-mandated interventions (including pre-trial diversion programs) for domestic violence offenders that target, in part or exclusively, batterers with the aim of reducing their future likelihood of re-assaulting above and beyond what would have been expected by routine legal procedures’.^50^	1986–2003	10 (4; 6; 0)	10 (batterer intervention movement developed from feminist movement)	7	Critically low	No effect
Gibbs *et al*^61^	‘We focus exclusively on HIV prevention interventions that combined economic empowerment interventions with gender-transformative interventions. The assumption underpinning these interventions is that men and women require a certain level of economic autonomy to enable them to act in more gender equitable ways’.^61^	NR	10 (6; 2; 2)	3	4, 6, 7	Critically low	Inconclusive/ mixed
Haberland^62^	‘To determine what existing evaluations of interventions for young people might suggest (regarding sexuality education programs and their inclusion of content on gender and power)’.^62^	1990–2012	27 (15; 0; 7)	9	1, 4, 6	Critically low	Positive effect
Harrison *et al*^63^	‘To inform the development of an evidence-based, state-of- the-art approach to youth HIV prevention in South Africa, we undertook a review of ongoing or recently completed intervention studies, with the aim of systematically assessing characteristics of rigourously designed youth HIV prevention interventions, to better under- stand how they work, and why’.^63^	2000 onwards (end date NR)	8 (4; 4; 0)	2	4, 6, 7	Critically low	Positive effect
Hartmann *et al*^41^	‘This review was conducted to inform a larger research priority setting exercise that is being undertaken to identify what research should be prioritised to strengthen the integration of effective gender equality interventions and human rights approaches in SRH programmes and policies’.^41^	1994–2014	33 (NR; NR; NR)	33 reviews included	1, 2, 4, 6	N/A	Inconclusive/ mixed
Heise^64^	‘This review focuses on a single form of violence—that which is perpetrated by intimate male partners. Examines the evidence base of a different topic potentially important to the prevention of partner violence’.^64^	NR	NR (NR; NR; NR)	NR	7	Critically low	Inconclusive/ mixed
Jennings *et al*^65^	‘We report the results of a comprehensive and systematic review of youth and young adult dating/intimate partner violence as well as reviewing interventions aimed at reducing such violence among individuals ages 15–30’.^65^	1981–2015	169 (34; 8; 127)	8 (two unknown if RCT/quasiexperimental)	6, 7	Critically low	Inconclusive/ mixed
Jewell and Wormith^66^	‘This meta-analysis explored the extent to which various demographic, violence-related, and intrapersonal variables were able to distinguish between treatment completers and dropouts’.^66^	1985–2010	30 (0; 90%; 3)	8	7	Critically low	Inconclusive/ mixed
Keleher and Franklin^67^	‘To identify the research evidence on programmatic interventions at the level of household and community that have been effective in changing gendered norms’.^67^	NR	NR (NR; NR; NR)	NR	6, 7	Critically low	Inconclusive/ mixed
Kraft *et al*^48^	Not explicitly reported: ‘Evidence-based behavior change interventions addressing gender dynamics must be identified and disseminated to improve child health outcomes’.^48^	NR	23 (NR; NR; NR)	Can't tell – 13 Gender-transformative for men	1, 2, 4, 6, 7	Critically low	Inconclusive/ mixed
Krishnaratne *et al*^42^	‘In this paper, we review the available evidence for HIV prevention as reflected in systematic reviews of HIV prevention interventions published during the past 20 years’.^42^	1995–2015	292 (90; NR; 137; additional 65 unaccounted for)	NR	4, 6	N/A	Inconclusive/ mixed
Lacroix *et al*^68^	‘To extend previous reviews by including more recent literature, determining the efficacy of couple-based HIV interventions in increasing condom use with both main and concurrent partners, and identifying moderators of intervention efficacy. We also explored commonly identified predictors of behaviour change as identified by past reviews (eg, provision of behavioural skills training) and included couple-specific moderators’.^68^	Up until mid-2012 (not stated in paper – based on supplemental data of when searches were run)	22 (NR; NR; NR)	NR	4	Critically low	Positive effect
Lopez *et al*^46^	‘This project systematically reviewed randomised controlled trials (RCTs) that examined the effect of theory-based interventions on contraceptive use’.^46^	NR	14 (14; 0; 0)	1	1, 4, 6	Critically low	Inconclusive/ mixed
McCloskey *et al*^69^	‘In this review, we will include studies which focus on one or several intersecting pathways to perpetration, the impact of IPV, and the efforts to prevent or end it’.^69^	1994 onwards (end date NR)	7 (4; 3; 1)	7	4, 6, 7	Critically low	Inconclusive/ mixed
Muralidharan *et al*^70^	‘1. To assess the extent to which gender-integrated health program in LMICs accommodate or transform gender norms, roles, and relationships; 2. To identify gender-accommodating and gender-transformative strategies in health program in LMICs (as defined in figure 1) 3. To understand how gender-integrated programs impact RMNCH+A, HIV, AIDS, GBV, TB, and UHC outcomes 4. To identify the quantitative and qualitative methods used to evaluate gender-integrated health programs’.^70^	NR	145 (25; 57; 64* study numbers reported do not add up)	Unclear – only proportions presented in graphs/charts	1, 2, 4, 6, 7	Critically low	Positive effect
Napierala Mavedzenge *et al*^71^	‘This updated review will focus on interventions carried out and/or published from January 2005 - December 2008. Since the first Steady, Ready, Go! (SRG) review was carried out, the results of several major randomised controlled trials of adolescent HIV prevention interventions conducted in Africa have been reported’.^71^	1990–2008	40 (23 studies) (11; 12; 0)	4	4, 6	Critically low	Inconclusive/ mixed
Rankin *et al*^72^	‘The aim of this EGM is to identify, map and describe existing empirical evidence and gaps in evidence on the effects SRH programming on adolescents in L&MICs. Our broader goal is to identify priorities for new impact evaluation and systematic review research’.^72^	1990 onwards (end date NR)	166 (101; 65; 0)	82	1, 4, 6, 7	Critically low	Inconclusive/ mixed
Rees *et al*^73^	‘This review aimed to summarise the current state of knowledge regarding health sector-based interventions for IPV, their integration into health systems and the perspectives of service users and healthcare workers on IPV care, focusing on the South African context’.^73^	Search conducted 2012–2014 – predetermined date range NR	NR (NR; NR; NR)	NR	6, 7	Critically low	Inconclusive/ mixed
Remme *et al*^74^	‘To systematically review evidence on the costs and cost-effectiveness of effective gender-responsive HIV interventions. In addition, where this has not been done, it seeks to explore the incremental cost and effects of gender-responsive programme components’.^74^	1990–2014	36 (19; 5; 12)	4	4, 6, 7	Critically low	Inconclusive/ mixed
Ricardo *et al*^75^	‘To investigate the effectiveness of interventions for preventing boys’ and young men’s use of sexual violence, including: increasing gender equitable attitudes, bystander intentions, and other attitudes and behaviors. It aims to explore the potential for intervening directly with boys and young men in community and school settings to address risk factors for sexual violence within diverse socio-cultural settings’.^75^	NR	65 (14; 51; 0)	65.* Only moderate and high quality study details reported	6, 7	Critically low	Positive effect
Sarkar *et al*^47^	‘This paper systematically reviews the effectiveness of interventions delivering maternal health services to young married women that include antenatal care, delivery care, postnatal care, contraception and safe abortion’.^47^	NR	8 (1; 3; 4)	1	1, 4, 6	Critically low	Positive effect
Schriver *et al*^76^	‘The current paper aims to: (1) describe the methodological approaches used to evaluate the impact of gender-integrated programmes on health outcomes in lower- and middle-income countries (LMICs); (2) identify and assess patterns in evaluation methods used in such programmes; and (3) provide recommendations for improving future evaluations of gender-integrated programmes in LMICs’.^76^	2008–2013 (interventions addressing RMNCH +A, HIV and AIDS, STIs, and GBV); 2000–2013 (interventions on TB, UHC, and health and nutrition of children ages 5 years and under)	99 (NR; NR; NR)	Not reported as numbers – 42% of gender-transformative studies used experimental design	1, 2, 4, 6, 7	Critically low	Inconclusive/ mixed
Skevington *et al*^77^	‘We report the first independent quantitative SR of evidence on the effectiveness of the Stepping Stones intervention’.^77^	1999–2010	8 (8; 0; 0)	7	4, 6	Critically low	Positive effect
Small *et al*^45^	‘To describe the range of interventions that incorporate gender-based content as a component of HIV and HIV risk interventions; to assess the methodological rigor of the evidence supporting these interventions and to assess the effectiveness of these interventions in reducing HIV-related risk behaviors and in reducing gender based violence’.^45^	1990–2012	11 studies (eight distinct interventions) (7; 2; 2)	3	4, 6	Critically low	Positive effect
Smedslund *et al*^78^	‘To assess if cognitive behavioural therapy (CBT) reduces violence from men who are physically violent towards their female partners’.^78^	inception-2010	12 (six individual trials)(12; 0; 0)	12	7	High	Inconclusive/ mixed
Storer *et al*^79^	‘Review question: 1. What are the goals, intervention components, and target audiences of bystander programs to prevent dating abuse and sexual violence? 2. What are the stated outcomes of evaluated bystander programs and what is the evidence of program efficacy at achieving these outcomes?’^79^	NR	16 (1; 1; 14)	2	6, 7	Critically low	Positive effect
Tokhi *et al*^49^	‘What interventions used to increase male involvement have been effective in increasing care-seeking behaviour during pregnancy, for childbirth and after birth for women and newborns and in improving key maternal and newborn health outcomes?’^49^	2000–2012	13 (3; 4; 6)	7	2	Critically low	Inconclusive/ mixed

*WHO domain (1–7): 1. Helping people realise their desired family size; 2. Ensuring the health of pregnant women and girls and their new-born infants; 3. Preventing unsafe abortion; 4. Promoting sexual health and well-being; 5. Promoting sexual and reproductive health in disease outbreaks; 6. Promoting healthy adolescence for a healthy future and unsafe abortion; harmful traditional practices, child, early and forced marriage; and sexual coercion and intimate partner violence; 7. Preventing and responding to violence against women and girls and harmful practices.

RCT, randomised controlled trial.

Although a number of reviews addressed outcomes spanning multiple domains, reviews were categorised into each WHO domain based on their primary outcome. The most commonly combined interventions addressed HIV and VAWG.^24 44 45^ The prevention of VAWG was the primary outcome most studied in reviews including gender-transformative interventions (46.2%, n=18). In contrast to the EGM, where promoting sexual health and well-being, was most frequently reported, a much smaller number of reviews of gender-transformative interventions reported on this outcome (23.1%, n=9) but, nonetheless, was the second most studied outcome.

Few reviews specifically disaggregated for outcomes related to male adolescent SRH. Within helping people realise their desired family size, only two reviews included interventions focusing on contraception,^46 47^ and no reviews of gender-transformative interventions were identified relating to (in)fertility. Two reviews examined the impact of engaging men/boys in gender-transformative interventions on maternal and new-born health.^48 49^ Finally, consistent with the EGM of total review evidence, no reviews of gender-transformative interventions were identified for which the primary outcome was preventing unsafe abortion or SRH in disease outbreaks (table 3).

**Table 3:** Number of reviews of gender-transformative interventions covering each WHO SRHR domain

WHO SRHR domain	Primary SRHR outcome covered in reviews (n of 39, % of reviews)*
1. Preventing and responding to violence against women/girls	18 (46.2)
2. Promoting sexual health and well-being	9 (23.1)
3. Promoting healthy adolescence for a healthy future	4 (10.3)
4. Helping people realise their desired family size	2 (5.1)
5. Health of pregnant women/girls and their new-born infants	2 (5.1)
6. Preventing unsafe abortion	0 (0)
7. Sexual and reproductive health in disease outbreaks (ie, Ebola and Zika)	0 (0)

*Additional category created for synthesis: *Promotion of Gender Equality and Resulting SRHR Outcomes* (n=4, 10.3%).

SRHR, sexual and reproductive health and rights.

Evidence of effectiveness is largely inconclusive yet promising. The majority of reviews reported mixed or inconclusive results relating to the effectiveness of engaging men/boys through gender-transformative approaches in SRHR (table 4). However, a third of reviews reported positive or promising outcomes, and only one review reported no effect.^50^ While no adverse effects were reported on SRHR outcomes in engaging men through gender-transformative approaches, two reviews in maternal and new-born health^48 49^ cautioned that the impact of some of these interventions on women’s autonomy remained ambiguous. This was especially true where health professionals and fathers were more educated than mothers underlying the imperative to examine for unintended effects on generating gender equalities.

**Table 4:** Concluded direction of results from included reviews for a gender-transformative approach to sexual and reproductive health interventions (n=39)

Inconclusive/mixed	23 (59%)
Positive effect	15 (38.5%)
No effect	1 (2.6%)

Table 2 and online supplementary file 1 identify each review conclusion and details of their included interventions.

Overall, however, the quality of evidence on effectiveness is limited for several reasons, including lack of critical mass of high-quality experimental gender-transformative intervention studies and limited studies including behavioural (eg, VAWG rates) or biological (eg, HIV status) outcomes. More of the included studies relied on outcomes based on self-reported attitudinal and norm changes and were measured over a limited time period (ie, under 1-year duration), which do not necessarily correlate or translate into behaviour change outcomes.

## Discussion

To our knowledge, this is the first comprehensive EGM reporting evidence on engaging men/boys from across the range of SRHR topics considered under the WHO reproductive health strategy.^33^ It is also the first systematic review of the impact of engaging men/boys through a gender-transformative approach on SRHR outcomes. The EGM highlights that while the majority of review evidence on male engagement lies in areas of sexual health, family planning and adolescent SRHR outcomes, there is very limited review evidence on topics related to maternal and child health, VAWG, unsafe abortion and SRH in disease outbreaks. Geoeconomically, the majority of the interventions engaging men/boys included in reviews is in LMICs.

The findings with most significant concerns for policy, research and programming relate to the limited number of reviews that include intervention studies that are gender-transformative (ie, that address harmful masculinities, male privilege over women or unequal power relations between women and men). The only outcome where the ratio of gender-transformative to non-gender-transformative reviews is approaching 1:1, and which a majority of the gender-transformative reviews cover, is in VAWG, which highlights the need for intentional and explicit promotion of gender equality and gender-transformative programming with men/boys.

The lack of gender-transformative work engaging men/boys, particularly in the area of SRHR, is a concern for a number of reasons. First, engaging men/boys in SRHR without explicit attention to gender inequalities can, at worst, be harmful particularly when it comes to undermining women’s rights and autonomy, or even where it is neutral or blind to these realities, can continue to perpetuate the status quo of gender inequalities. Second, as this review shows, the assumption that engaging men/boys in SRHR in and of itself can promote gender equality is false and needs to be challenged. Closer examination is required of the premise/aim of the intervention, the theory of change and whether there is explicit attention to issues of male privilege, power and positionality in relation to women.

Moreover, almost 25 years after the ICPD call for male engagement as an approach to promoting gender equality, the findings of this review highlight that the evidence remains sparse in terms of rigour and quality and in demonstrating conclusively the effectiveness on a range of SRHR outcomes. Encouragingly, approximately 40% of the reviews containing gender-transformative interventions showed positive findings on one or more outcomes and few showed negative outcomes. However, findings should be interpreted with caution in light of low-quality review evidence. This highlights that with more rigorous study designs and outcome measures used, as well as attention to programme and evaluation quality and reporting, progress is likely.

## Conclusion

Analysis of the review evidence provides direction for a strengthened research agenda. First, there is a need to strengthen programme reporting standards when it comes to reviews and studies—as it is obvious from table 2 that many of the parameters were not reported or unclear while extracting data. Second is the need for future studies to go beyond self-reported attitudinal outcomes by men and include more biobehavioural outcomes. Third is the need for evaluations to have a longer period of time for programme effects to show results downstream. Fourth is the need for programme implementers and researchers to be explicit about the pathways by which change is likely to occur. Finally, the limited number of higher quality intervention studies (ie, quasi-experimental or RCT design), particularly those gender-transformative in nature, highlights the need for investment in more rigorous approaches.

A number of limitations of this review warrant acknowledgement. A general limitation of a review of reviews is there is a risk of missing newer evidence from interventions that have not yet been included in systematic reviews.^51^ Although language was not a limit applied, no non-English language reviews of gender-transformative interventions were identified, possibly a result of only English language search terms used. The focus on effectiveness limited our selection to experimental and quasi-experimental studies, omitting cross-sectional and solely qualitative studies.

In conclusion, the review demonstrates we have not yet reached a tipping point in gender-transformative work with men/boys to improve SRHR outcomes. The next generation of investments in research and programming on male engagement needs to consolidate this emerging evidence and assess SRHR outcomes that are less well covered such as maternal and new-born health, family planning, safe abortion, infertility and SRH in disease outbreaks. Research and programming needs to be intentional in promoting gender equality and monitoring any adverse or unexpected outcomes that may result from interventions. Gender-transformative programming requires a balance between appealing to men in order to effectively engage with them and challenging men to contest gender inequalities.^52^ Efforts should focus on exploring the characteristics of interventions where promising or positive results were found in order to further unpack what approaches to male engagement with gender-transformative programming are likely to be most effective, the pathways of change and the types of outcomes that can provide better measures of what works. Furthermore, triangulation with qualitative data highlighting where and how change might have taken place in men’s attitudes and behaviours is important. This requires greater partnership between programme implementers and researchers in order to realise the potential for engaging men/boys in promoting gender equality for SRHR.
