# Supplementary material for: Interventions addressing men, masculinities and gender equality in sexual and reproductive health and rights: an evidence and gap map and systematic review of reviews
Source: BMJ Glob Health. 2019 Sep 11;4(5):e001634. doi: 10.1136/bmjgh-2019-001634 (PMC6747894; doi:10.1136/bmjgh-2019-001634)
Supplement: Supplementary data [file bmjgh-2019-001634supp001.pdf]

Appendices

Appendix 1. Included reviews categorised by intentionality of gender-transformative intervention inclusion

**Category A** *Sought to include gender-transformative interventions*	**Category B** *Did not seek to include gender-transformative interventions, yet at least one gender-transformative intervention was included.*
(Bacchus et al., 2017)	(Anderson et al., 2013)
(Casey et al., 2018)	(Arango et al., 2014)
(Chatterjee, 2015)	(Arias et al., 2013)
(Dworkin et al., 2013)	(Bakrania et al., 2017)
(Ellsberg et al., 2015)	(Bourey et al., 2015)
(Feder et al., 2008)	(DeGue et al., 2014)
(Gibbs et al., 2012)	(Denison et al., 2009)
(Haberland, 2015)	(Harrison et al., 2010)
(Hartmann et al., 2016)	(Jennings et al., 2017)
(Heise, 2011)	(Krishnaratne et al., 2016)
(Jewell & Wormith, 2010)	(Lacroix et al., 2013)
(Keleher & Franklin, 2008)	(Lopez et al., 2009)
(Kraft et al., 2014)	(McCloskey et al., 2016)
(Muralidharan et al., 2015)	(Napierala Mavedzenge et al., 2010)
(Remme et al., 2014)	(Rankin et al., 2016)
(Ricardo et al., 2011)	(Rees et al., 2014)
(Schriver et al., 2017)	(Sarkar et al., 2015)
(Small et al., 2013)	(Skevington et al., 2013)
(Tokhi et al., 2018)	(Smedslund et al., 2011)
	(Storer et al., 2015)

Ruane-McAteer E, *et al. BMJ Global Health* 2019; 4:e001634. doi: 10.1136/bmjgh-2019-001634

Appendix 2. Search strategy

Search terms related to SRHR were adapted from Warren et al (2015) with the addition of "maternal mortality", "forced sex" "sexual slavery", "sexual exploitation" "coercive control", "child prostitut*", "child trafficking", "trafficking of child*", "female genital mutilation", "FGM", "female genital cutting", "FGMC", "female circumcis*", "fertile*", "infertil*", (early and marriage), (child and marriage), (forced and marriage), (arranged and marriage), (abduction and marriage). Terms related to FGM and child marriage were adapted from Greene (2014) and Karumbi et al (2017). A number of more generic terms not specifically related to SRHR were removed from Warren et al (2015) string (e.g. "violence" "physical assault").

Terms related to males and masculinities were developed and tested in a number of databases to ensure they captured all relevant papers. An edited Pearl Harvesting approach to searching databases for systematic reviews was utilised to identify systematic review papers (Sandieson, 2006). Two terms were removed from Sandieson's original Systematic Review search string due to them producing a large number of irrelevant articles ("qualitative synthesis" and "realist synthesis"). While the Pearl Harvesting approach produced a large number of search results, after testing a number of more simplified searches for systematic reviews it was found that a number of potentially relevant articles would be missed without it. Search terms related to trials were adapted from Cochrane approved guidance (Eady, Wilczynski, & Haynes, 2008; Watson & Richardson, 1999). SRHR, men and masculinities, systematic review, and trial search strings were combined and tested in three key databases (Medline, PsycINFO, Embase) before final agreement for terms was reached.

**Search terms used**	
Search combination: #1 AND #2 AND #3 AND #4 (limited to Human; 2007-present)	
#1 SRHR	sexual health or reproductive health or maternal health or maternal welfare or maternal mortality or neonatal health or perinatal care or perinatal health or prenatal care or prenatal health or antenatal health or ante-natal health or postnatal health or post-natal health or post-part* or post part* or newborn health or family planning or contracepti* or condoms or condom or pregnan* or abortion or induced abortion or abort* or birth or miscarriage or spontaneous abortion or stillb* or Minimum Initial Service Package or obstetric* or gynecology or gynaecology or safe motherhood or safe delivery or skilled birth attend* or sexually transmitted infection* or sexually transmitted disease* or HIV or Human immunodeficiency virus or AIDS or acquired immune deficiency syndrome or PMTCT or rectovaginal fistula or urethra fistula or urinary tract fistula or genital trauma or genital injury or vaginal trauma or vaginal injury or gender-based violence or gender based violence or partner violence or family violence or violence against women or domestic violence or sexual abuse or sex crime or sexual crime or domestic violence or sexual violence or rape or intimate partner violence or partner violence or partner abuse or sexual assault or sexual harassment or sexual coercion or forced sex or sexual slavery or sexual exploitation or coercive control or child prostitut* or child trafficking or trafficking of child* or female genital mutilation or FGM or female genital cutting or FGMC or female circumcis* or fertile* or infertil* or (early and

	marriage) or (child and marriage) or (forced and marriage) or (arranged and marriage) or (abduction and marriage)
#2 Males/ Masculinities	men or man or male or males or boy or boys or masculin* or father* or gender or equality
#3 Systematic Review	"data synthesis" or "evidence synthesis" or metasynthesis or meta-synthesis or "narrative synthesis" or "quantitative synthesis" or "research synthesis" or "synthesis of evidence" or "thematic synthesis" or metaanaly* or meta-analy* or metaanalysis or meta-analysis or systematic or "systematic map*" or "systematic overview*" or "systematic review*" or "systematically review*" or "bibliographic search" or "database search" or "electronic search" or handsearch* or "hand search*" or "keyword search" or "literature search" or "search term*" or "article reviews" or "literature review" or "overview of reviews" or "review literature" or "reviewed the literature" or "reviews studies" or "this review" or "scoping stud*" or "overview study" or "overview of the literature" or meta-ethnograph* or meta-epidemiological or "data extraction" or "meta-regression" [title only]
#4 Trials	random* or trial or placebo or group or groups or intervention or interventions

Appendix 3. Evidence and Gap Map Screening and Categorisation form

1. Is this paper a systematic review about sexual and reproductive health interventions and including men? [Systematic Review = systematic search used]
   Yes No Can't tell

2. Does this systematic review include RCT (experimental with randomisation) or quasi-experimental
   Yes No Can't tell

3. What area of WHO Reproductive Health Strategy do the outcomes of the review fall under? (checkbox)
   a. Helping people realise their desired family size (i.e. contraception and family planning; prevention and treatment)
   b. Health of pregnant women and girls and their newborn infants (i.e. maternal and infant mortality)
   c. Preventing unsafe abortion
   d. Promoting Sexual Health and Wellbeing (i.e. prevention of reproductive tract and sexually transmitted infection
   e. Sexual and Reproductive Health in disease outbreaks (i.e. Ebola and Zika)
   f. Healthy Adolescence for a Healthy Future (i.e. any of the areas covered in other domains focusing on adolescent
   g. Preventing and responding to violence against women and girls (i.e. IPV, sexual violence) and harmful practice
   h. Can't tell if above ticked area(s) is exhaustive list
   i. Can't tell what areas covered None of the above

4. Does the review seek to distinguish studies that are gender-transformative?

["Gender-transformative" is defined by the World Health Organization (WHO) as programmes '**that addresses the causes of gender-based health inequities by including ways to** transform harmful gender norms, roles and relations. The objective of such programmes is often to promote gender equality between women and men' (WHO, 2011: 78). In our case we are looking for gender-transformative approaches with men – so they have to engage men in transforming either a. gender norms (masculinities); or b. gender roles (men's practices); or c. gender relations (unequal relations between men and women) to achieve sexual and reproductive health outcomes. The review should state whether or not it included, or wholly focussed on, gender-transformative approaches to outcomes.]

Yes No Can't tell

5. Were the interventions delivered in high, middle, or low income countries?
Low Middle High Can't tell

Ruane-McAteer E, *et al. BMJ Global Health* 2019; 4:e001634. doi: 10.1136/bmjgh-2019-001634

Appendix 4. Systematic Review of Reviews Data Extraction form (Post-pilot version)

1. What area of WHO Reproductive Health Strategy does this review fall under? (Select checkbox and state area review focuses on)
    a. Helping people realise their desired family size (i.e. contraception and family planning; prevention and treatment of infertility)
    b. Health of pregnant women and girls and their newborn infants (i.e. maternal and infant mortality; preventing complications in pregnancy, childbirth and the postnatal period)
    c. Preventing unsafe abortion
    d. Promoting Sexual Health and Wellbeing (i.e. prevention of reproductive tract and sexually transmitted infections; HIV; sexuality related human rights abuses (e.g. sexual coercion)
    e. Sexual and Reproductive Health in disease outbreaks (i.e. Ebola and Zika)
    f. Healthy Adolescence for a Healthy Future (i.e. any of the areas covered in other domains focusing on adolescents only)
    g. Preventing and responding to violence against women and girls (i.e. IPV, sexual violence) and harmful practices (i.e. FGM; early child forced marriage)
    h. Can't tell
2. Date range of search
3. Documented search strategy
    a. Search terms reported (y/n)
    b. Location of search specified (databases) (y/n)
    c. Inclusion/Exclusion criteria applied (detail)
    d. Language restrictions (detail)
    e. Other search detail (detail)
4. Number of studies reviewed
5. Review Aims/Objectives
6. Were the interventions delivered in high, middle, or low income countries?
    a. Low
    b. Middle
    c. High
    d. Not reported
7. Types of studies included (RCT n=x; quasi-experimental n=y; other study design n=z)
    a. RCTs (number)
    b. Quasi-Experimental (number)
    c. Other study designs (number)
    d. Not reported (detail)
8. Number of gender-transformative experimental studies (quasi-experimental/RCT)
9. Included gender-transformative intervention detail
    a. Programme name/description; population
    b. Men only (y/n)
    c. Community level (y/n)
    d. WHO domain (1-7)
    e. Country (name)
    f. Study design
    g. No of participants
    h. Participants (m/f)
    i. GT aim of review or aim of included stud(ies)
    j. Review outcomes & conclusions
    k. Review limitations

Ruane-McAteer E, *et al. BMJ Global Health* 2019; 4:e001634. doi: 10.1136/bmjgh-2019-001634

Appendix 5. Proportion of reviews included in EGM reporting on interventions using a gender-transformative approach

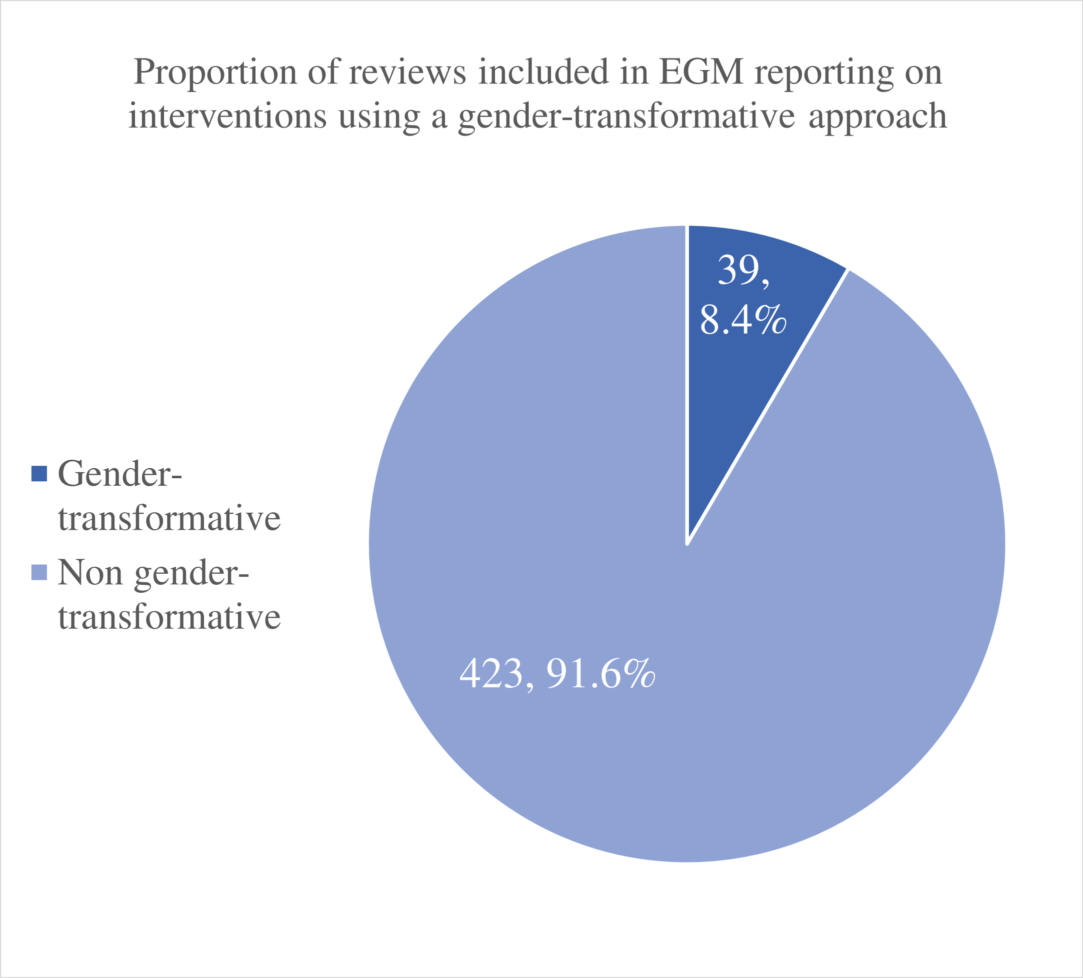

Appendix 6
AMSTAR2 Quality rating of included reviews[1]

Study	AMSTAR 2 Rating																
	**Q1**	**Q2**	**Q3**	**Q4**	**Q5**	**Q6**	**Q7**	**Q8**	**Q9**	**Q10**	**Q11**	**Q12**	**Q13**	**Q14**	**Q15**	**Q16**	**Overall rating**
Anderson 2013	N	N	N	N	N	N	N	N	N	N	NC	NC	N	N	NC	Y	Critically low
Arias 2013	N	N	N	P	N	Y	N	N	N	N	Y	N	N	N	NC	Y	Critically low
Bacchus 2017	N	N	N	N	N	N	N	Y	N	N	NC	NC	N	N	NC	Y	Critically low
WHO 2007	N	N	N	N	N	Y	N	P	N	N	NC	NC	N	N	NC	N	Critically low
Bourey 2015	N	Y	N	P	Y	Y	N	P	Y	N	NC	NC	Y	N	NC	Y	Critically low
Casey 2018	N	N	N	N	N	N	N	N	N	N	NC	NC	N	N	NC	Y	Critically low
Chatterjee 2015	Y	N	N	N	N	N	N	P	N	N	NC	NC	N	N	NC	N	Critically low
DeGue 2014	N	N	Y	N	N	Y	N	P	N	N	NC	NC	Y	N	NC	N	Critically low
Denison 2009	Y	N	N	Y	Y	Y	Y	Y	Y	Y	Y	Y	Y	Y	N	N	Low

[1] The AMSTAR2 tool (Shea et al., 2017) was used to assess the methodological quality of these systematic reviews. This version of the original AMSTAR tool was developed specifically for the purposes of assessing quality of systematic reviews reporting on interventions that utilised either randomised or non-randomised study designs in healthcare settings. In accordance with guidance by Shea et al. (2017), the overall rating represented the reviewers' overall confidence in the results of each review. A high quality rating was assigned if no or one non-critical weakness was identified, moderate quality with more than one non-critical weakness, low quality with one critical flaw and critically low quality with more than one critical flaw. Reasons for included reviews being rated as critically low were the identification of more than one major methodological weaknesses, such as (i) not reporting explicitly that the review methods were established prior to the conduct of the review with a registered protocol; (ii) an inadequate search of the literature; (iii) no justification for excluding individual studies; (iv) no assessment of the risk of bias from individual studies included; (v) no consideration of risk of bias when interpreting results from the review; and/or (vi) no assessment of publication bias.

Study	AMSTAR 2 Rating																
	**Q1**	**Q2**	**Q3**	**Q4**	**Q5**	**Q6**	**Q7**	**Q8**	**Q9**	**Q10**	**Q11**	**Q12**	**Q13**	**Q14**	**Q15**	**Q16**	**Overall rating**
Dworkin 2013	N	N	N	N	N	N	N	P	N	N	NC	NC	N	N	NC	N	Critically low
Ellsberg 2015	N	N	N	P	Y	Y	N	N	N	N	NC	NC	Y	Y	NC	Y	Critically low
Feder 2008	Y	N	N	P	Y	Y	P	P	P	N	N	N	Y	Y	N	Y	Critically low
Gibbs 2012	N	N	N	P	N	N	N	N	N	N	NC	NC	N	N	NC	Y	Critically low
Haberland 2015	N	N	N	N	N	Y	N	N	N	N	NC	NC	N	Y	NC	N	Critically low
Harrison 2010	Y	N	N	P	N	N	N	P	N	N	NC	NC	N	N	NC	Y	Critically low
Heise 2011	N	N	N	N	N	N	N	P	N	N	NC	NC	N	N	NC	Y	Critically low
Jennings 2017	N	N	N	N	N	N	N	N	N	N	NC	NC	N	N	NC	N	Critically low
Jewell 2010	N	N	N	N	N	Y	Y	P	N	N	Y	N	N	Y	N	N	Critically low
Keleher 2008	N	N	N	N	N	N	N	N	N	N	NC	NC	N	N	NC	N	Critically low
Kraft 2014	N	N	N	N	N	N	N	N	N	N	NC	NC	N	N	NC	Y	Critically low
LaCroix 2013	Y	N	N	P	N	Y	N	N	N	N	Y	N	N	N	Y	Y	Critically low
Lopez 2009	Y	N	N	Y	Y	Y	N	N	P	N	NC	NC	N	N	NC	Y	Critically low
Mavedzenge 2010	Y	N	N	Y	Y	N	N	P	N	N	NC	NC	N	N	NC	Y	Critically low
McCloskey 2016	N	N	N	N	N	N	N	P	N	N	NC	NC	N	N	NC	N	Critically low

Ruane-McAteer E, *et al. BMJ Global Health* 2019; 4:e001634. doi: 10.1136/bmjgh-2019-001634

Study	AMSTAR 2 Rating																
	**Q1**	**Q2**	**Q3**	**Q4**	**Q5**	**Q6**	**Q7**	**Q8**	**Q9**	**Q10**	**Q11**	**Q12**	**Q13**	**Q14**	**Q15**	**Q16**	**Overall rating**
Muralidharan 2015	N	N	N	P	Y	Y	N	N	N	N	NC	NC	N	N	NC	N	Critically low
Rankin 2016	N	N	Y	Y	N	Y	N	N	N	N	NC	NC	N	N	NC	Y	Critically low
Rees 2014	N	N	N	P	N	N	N	N	N	N	NC	NC	N	N	NC	Y	Critically low
Remme 2014	N	N	N	N	N	Y	N	P	N	N	NC	NC	N	N	NC	Y	Critically low
Ricardo 2011	Y	N	Y	P	N	N	N	P	Y	N	NC	NC	Y	N	NC	N	Critically low
Sarkar 2015	N	N	N	N	Y	Y	Y	N	N	N	NC	NC	N	N	NC	Y	Critically low
Schriver 2017	N	N	N	N	N	N	N	N	N	N	NC	NC	N	N	NC	Y	Critically low
Skevington 2013	Y	N	Y	P	N	Y	N	P	N	N	NC	NC	N	N	NC	N	Critically low
Small 2013	N	N	N	N	Y	Y	N	P	P	N	NC	NC	N	N	NC	N	Critically low
Smedslund 2011	Y	Y	Y	Y	Y	Y	Y	Y	Y	N	Y	Y	Y	Y	Y	Y	High
Storer 2015	N	N	N	N	N	N	N	P	N	N	NC	NC	N	N	NC	Y	Critically low
Tokhi 2018	Y	Y	Y	P	Y	Y	N	N	Y	N	NC	NC	N	N	NC	Y	Critically low

Abbreviations: Yes (Y), No (N), Partial Yes (P), Not Conducted (NC)

Appendix 7
Gender-transformative interventions included in each review[2]

Review author(s) Conclusion: Positive Effect No Effect Inconclusive / Mixed	Intervention author(s)	Year	Programme name/description	Delivery*		WHO Domain (1-7) †	Country	Design	No of Participants	Participants m/f	Intervention objective: gender-transformative Aim/By-product
				Men only	Community level						
**Anderson *et al.* 2013** Positive Effect	Jewkes et al	2008	Stepping Stones	No	No	1,4,6,7	South Africa	RCT	2776	NR	Aim
**Arias *et al.* 2013** Inconclusive / Mixed	Saunders	1996	Duluth + CBT	NR	NR	7	NR	Quasi	129	NR	Aim
	Dobash *et al.*	1996	Duluth	NR	NR	7	NR	Quasi	40	NR	Aim
	Murphy *et al.*	1998	Duluth	NR	NR	7	NR	Quasi	235	NR	Aim
	Babcock & Steiner	1999	Duluth	NR	NR	7	NR	Quasi	106	NR	Aim
	Jones & Gondolf	2002	Duluth	NR	NR	7	NR	Quasi	853	NR	Aim
	Bowen *et al.*	2005	Duluth	NR	NR	7	NR	Quasi	86	NR	Aim
	Bennet *et al.*	2005	Duluth	NR	NR	7	NR	Quasi	384	NR	Aim
	Labriola *et al.*	2005	Duluth	NR	NR	7	NR	Quasi	157	NR	Aim
	Tollefson & Gross	2006	Duluth	NR	NR	7	NR	Quasi	102	NR	Aim
	Coulter & Van de Weerd	2009	Duluth	NR	NR	7	NR	Quasi	12522	NR	Aim
	Davis *et al.*	1998	Duluth	NR	NR	7	NR	RCT	376	NR	Aim
	Feder & Dugan	2004	Duluth	NR	NR	7	NR	RCT	404	NR	Aim
	Stith *et al.*	2004	Duluth + CBT	NR	NR	7	NR	RCT	39	NR	Aim
	Lin *et al.*	2009	Duluth + CBT	NR	NR	7	NR	RCT	301	NR	Aim

[2]Of the 226 gender-transformative intervention studies in which study characteristics of interest could be identified from reviews, the majority included males and females in the intervention studies (n=126, 55.8%), 71 (31.4%) were delivered to males only, and for 29 (12.8%) of these interventions this data could not be extracted from the reviews. Regarding intervention delivery, approximately a quarter was delivered at community level (n=63, 27.9%), half was delivered at individual or targeted group (n=115, 50.9%), and this data could not be extracted from reviews for 48 interventions (21.2%).

**Review author(s) Conclusion:** Positive Effect No Effect Inconclusive / Mixed	**Intervention author(s)**	**Year**	**Programme name/description**	**Delivery***		**WHO Domain (1-7) †**	**Country**	**Design**	**No of Participants**	**Participants m/f**	**Intervention objective: gender-transformative Aim/By-product**
				**Men only**	**Community level**						
	Taylor & Maxwell	2009	Duluth	NR	NR	7	NR	RCT	629	NR	Aim
**Bacchus *et al.* 2017** Positive Effect	Ashburn *et al.*	2016	Responsible, Engaged and Loving (REAL) Fathers	Yes	No	6,7	Uganda	RCT	250 intervention; 250 control	100% male	Aim
	Kyegombe *et al.*	2015	SASA!	No	Yes	6,7	Uganda	RCT	1538 (baseline); 2532 (4 year follow up) *not divided by intervention/control	NR	Aim
	Abramsky *et al.*	2014, 2016	SASA!	No	Yes	6,7	Uganda	RCT	Baseline: 793 (intervention), 790 (control); Four Year Follow up: 1368 (intervention), 1164 (control)	Intervention: n=374 women, n = 419 men Control: n = 343 women, n = 447 men. At four year follow up: Intervention communities: n = 600 women and n = 768 men and Control communities: n = 530 women and n = 634 men	Aim
**Bourey *et al.* 2015** Positive Effect	Green	2015	WING: Women's Income Generating Support (WINGS)	No	No	6,7	Uganda	Cluster RCT	1734	NR	NR
	Gupta	2013	Gender Dialogue Groups (GDG) for VLSA (Village Savings and Loans Association)	No	No	7	Cote d'Ivoire	Cluster RCT	913-934	NR	NR
	Jewkes *et al.*	2008	Stepping Stones	No	No	7	South Africa	Cluster RCT	1041-1093	537-559 Women/ 504-534 Men	NR
	Pulerwitz	2015	Male Norms Initiative	Yes	No	6,7	Ethiopia	Cluster RCT	645	645 Men	NR
**Casey *et al.* 2018** Positive Effect	Abramsky *et al.*	2014	SASA!	No	Yes	7	Uganda	NR	NR	100% male	Aim
	Hossain *et al.*	2014	"Men and Women in Partnership Initiative"	No	No	7	Cote d'Ivoire	NR	NR	NR	By-product
	Jewkes *et al.*	2014	"Stepping Stones and Creating Futures"	No	No	7	South Africa	NR	NR	NR	Aim
	Kalichman *et al.*	2009	"GBV/HIV versus Alcohol/HIV"	Yes	No	7	South Africa	NR	NR	NR	By-product
	Krishnan *et al.*	2012	"RESPECT"	No	No	7	Tanzania	NR	NR	NR	Aim
	Kyegombe *et al.*	2014	SASA!	Yes	Yes	7	Uganda	NR	NR	100% male	Aim

Review author(s) Conclusion: Positive Effect No Effect Inconclusive / Mixed	Intervention author(s)	Year	Programme name/description	Delivery*		WHO Domain (1-7) †	Country	Design	No of Participants	Participants m/f	Intervention objective: gender-transformative Aim/By-product
				Men only	Community level						
	Miller *et al.*	2014	"Parivartan— Adaptation of Coaching Boys into Men"	Yes	No	6,7	India	NR	NR	NR	Aim
	Pulerwitz *et al.*	2015	"Male Norms Initiative"	Yes	Yes	6,7	Ethiopia	NR	NR	100% male	Aim
	Salazar *et al.*	2014	"Real Consent"	No	No	7	United States	NR	NR	NR	Aim
	Wagman *et al.*	2015	"The Safe Homes and Respect for Everyone Project"	No	Yes	6,7	Uganda	NR	NR	100% male	By-product
**DeGue *et al.* 2014** Inconclusive / Mixed	Foshee *et al.*	1998, 2000, 2004, 2005	Safe Dates	NR	No	6,7	NR	RCT	NR (14 schools)	NR	Aim
	Gidycz *et al.*	2011	The Men's Project	Yes	No	6,7	NR	RCT	NR	NR	Aim
**Denison *et al.* 2009** Inconclusive / Mixed	Chege	2004	Education through behaviour change communication activities, and advocacy	No	Yes	1,7	Ethiopia and Kenya	Quasi	2259	1113 Male/ 1126 Female	Aim
	Diop	2004	Education in hygiene, problem solving, women's health, human rights	No	Yes	7	Senegal	Quasi	1332	557 Male/ 775 Female	Aim
	Ouoba	2004	Education in hygiene, problem solving, women's health, human rights	No	Yes	7	Burkina Faso	Quasi	1465	1047 Male/ 718 Female	Aim
	Babalola	2006	Community mobilization; advocacy; mass media activities	No	Yes	7	Nigeria	Quasi	957	426 Male/ 531 Female	Aim
**Dworkin *et al.* 2013**	Cupples *et al.*	2008	"M.A.R.S. Male Advocates for Responsible Sexuality"	No	Yes	6,7	USA	Quasi	300	53% male	Aim
	Jewkes *et al.*	2008	"Stepping Stones"	No	Yes	6,7	South Africa	RCT	1140 (intervention) 1081 (control)	Intervention (51% male); Control (50% male)	By-product

**Review author(s) Conclusion:** Positive Effect No Effect Inconclusive / Mixed	**Intervention author(s)**	**Year**	**Programme name/description**	**Delivery***		**WHO Domain (1-7) †**	**Country**	**Design**	**No of Participants**	**Participants m/f**	**Intervention objective: gender-transformative Aim/By-product**
				**Men only**	**Community level**						
Positive Effect	Kalichman *et al.*	2009	"Phaphama Men"	Yes	No	7	South Africa	Quasi	229 (intervention); 203 (control)	100% male	Aim
	Miller *et al.*	2012	"Coaching Boys into Men"	Yes	No	6,7	USA	RCT	847 (intervention); 951 (control)	100% male	Aim
	Pulerwitz *et al.*	2010	"Male Norms Initiative"	Yes	Yes	6,7	Ethiopia	Quasi	251 (arm 1); 235(arm 2); 159(arm 3)	100% male	Aim
	Pulerwitz *et al.*	2006	"Program H"	Yes	Yes	6,7	Brazil	Quasi	217 (arm1); 190(arm2); 180(arm3)	100% male	Aim
	Rhodes *et al.*	2011	HoMBReS-2 (hombres manteniedo bienstar y relaciones saludables-2/ men-2: men maintaining wellbeing and health relationships)	Yes	No	7	USA	RCT	58(intervention); 64(control	100% male	By-product
	Verma *et al.*	2008	"Yaari-Dosti"	Yes	No	7	India	Quasi	197(arm1); 472(arm2); 466(arm3)	100% male	Aim
**Ellsberg *et al.* 2015** Positive Effect	Jewkes *et al.*	2008	Stepping Stones	No	No	6,7	South Africa	RCT	NR	NR	Unclear
	Verma *et al.*	2008	Group 1. Received a lifestyle social marketing campaign and group education sessions (LSMC plus GES). Group 2. Received only the group education sessions. The third group was the control	Yes	No	6,7	India	Quasi	NR	100% male	Unclear
	Abramsky *et al.*	2014	SASA!	No	Yes	7	Uganda	RCT	NR	NR	Aim
	Diop *et al.*	2004	Tostan	No	Yes	6,7	Senegal	RCT	NR	NR	Aim
	Wagman *et al.*	2014	SHARE	No	Yes	6,7	Uganda	RCT	NR	NR	Unclear
	Duflo *et al.*	2006	Evaluation comparing 3 school-based HIV/AIDS interventions in Kenya	No	No	6,7	Kenya	RCT	70000 students from 328 primary schools	NR	Unclear
	Gupta *et al.*	2013	Both an 8 session 16 week gender dialogue group (GDG) and an economic empowerment	No	No	7	Cote d'Ivoire	RCT	NR	NR	Aim

**Review author(s) Conclusion:** Positive Effect No Effect Inconclusive / Mixed	**Intervention author(s)**	**Year**	**Programme name/description**	**Delivery***		**WHO Domain (1-7) †**	**Country**	**Design**	**No of Participants**	**Participants m/f**	**Intervention objective: gender-transformative Aim/By-product**
				**Men only**	**Community level**						
			group savings programme (VSLA) vs VSLA only								
	Haushofer *et al.*	2013	The Give Directly programme (unconditional cash transfers)	No	No	7	Kenya	RCT	1010 primary women households	NR	Unclear
	Hidrobo *et al.*	2013	Cash, Food, and Voucher Program; households received 6 monthly transfers of vouchers, cash, or food	No	No	6,7	Ecuador	RCT	NR	NR	Unclear
**Feder *et al.* 2008** No Effect	Chen *et al.*	1989	Cognitive-behavioural/ Psycho-educational	Yes	No	7	USA	Quasi	NR	NR	Aim
	Davis *et al.*	2000	Psycho-educational	Yes	No	7	USA	RCT	NR	NR	Aim
	Dunford	2000	Cognitive-behavioural	Yes	No	7	USA	RCT	NR	NR	Aim
	Dutton	1986	Cognitive-behavioural	Yes	No	7	USA	Quasi	NR	NR	Aim
	Feder & Forde	2000	Cognitive-behavioural/ Psycho-educational	Yes	No	7	USA	RCT	NR	NR	Aim
	Gordon & Moriarty	2003	Cognitive-behavioural/ Psycho-educational	Yes	No	7	USA	Quasi	NR	NR	Aim
	Harrell	1991	Cognitive-behavioural	Yes	No	7	USA	Quasi	NR	NR	Aim
	Jones & Gondolf	2002	Cognitive-behavioural	Yes	No	7	USA	Quasi	NR	NR	Aim
	Palmer *et al.*	1992	Psycho-educational	Yes	No	7	USA	RCT	NR	NR	Aim
	Syers & Edleson	1992	Psycho-educational	Yes	No	7	USA	Quasi	NR	NR	Aim
**Gibbs *et al.* 2012** Inconclusive / Mixed	Duflo *et al.*	2006	Western Kenya Schooling Intervention	No	No	6,7	Kenya	RCT	70000	NR	Unclear
	Ssewamala *et al.*	2010	SUUBI Research Programme	No	No	6,7	Uganda	RCT	277	NR	Unclear
	Hallman *et al.*	2011	Siyakha Nentsha Programme	No	No	6,7	South Africa	Quasi	NR	NR	Unclear

**Review author(s) Conclusion:** Positive Effect No Effect Inconclusive / Mixed	**Intervention author(s)**	**Year**	**Programme name/description**	**Delivery***		**WHO Domain (1-7) †**	**Country**	**Design**	**No of Participants**	**Participants m/f**	**Intervention objective: gender-transformative Aim/By-product**
				**Men only**	**Community level**						
**Haberland 2015** Positive Effect	Allen *et al.*	1997	Teen Outreach Programme	No	No	1,4,6	United States	RCT	695	15% male, 85% female	Aim
	Cowen *et al.*	2010	Regai Dzive Shiri: Youth programmes for schools (used MEMA Kwa Vijana curriculum, adding sessions on gender issues, communication, self-belief and self-awareness)	NR	Yes	1,4,6	Zimbabwe	RCT	4684	NR	Aim
	Cowen *et al.*	2008	See Cowen et al (2010) above.	NR	Yes	1,4,6	Zimbabwe	RCT	4684	NR	Aim
	Dupas	2011	Relative Risk Information Campaign	NR	No	1,4,6	Kenya	RCT	328	NR	Aim
	Jewkes *et al.*	2008	Stepping Stones: Adapted for South Africa	No	Yes	1,4,6	South Africa	RCT	2776	1360 male, 1416 female	Aim
	Philliber	2002	Children's Aid Society-Carrera Programme	No	Yes	1,4,6	United States	RCT	484	55% female, 45% male	Aim
	Ross *et al.*	2007	MEMA kwa Vijana	NR	Yes	1,4,6	Tanzania	RCT	9645	NR	Aim
	Osabi *et al.*	2006	MEMA kwa Vijana	NR	Yes	1,4,6	Tanzania	RCT	9645	NR	Aim
	Doyle *et al.*	2010	MEMA kwa Vijana	NR	Yes	1,4,6	Tanzania	RCT	9645	NR	Aim
**Harrison *et al.* 2010** Positive Effect	Exner *et al.*	2006	Mpondombili Project	No	No	4,6,7	South Africa	Quasi	442 (intervention); 541 (control)	NR	Aim
	Jewkes *et al.*	2006, 2008	Stepping Stones	No	Yes	4,6,7	South Africa	RCT	1140 (intervention); 1081 (control)	NR	Aim
**Heise 2011** Inconclusive / Mixed	Kim	2009	IMAGE	No	Yes	7	South Africa	Community RCT	NR	NR	Aim
	Jewkes (combined reporting)	2008 & 2010	Stepping Stones	No	No	1,7,4	South Africa	Cluster RCT	2776	1360 Male/1416 Female	Aim
	Verma	2008	Yaari Dosti	Yes	No	4,6,7	India	Quasi	1138	1138 Men	Aim
	Bradley *et al.*	2009	Structured Videos	No	No	6,7	USA	Unknown	309	113 male/196 females	NR

**Review author(s) Conclusion:** Positive Effect No Effect Inconclusive / Mixed	**Intervention author(s)**	**Year**	**Programme name/description**	**Delivery***		**WHO Domain (1-7) †**	**Country**	**Design**	**No of Participants**	**Participants m/f**	**Intervention objective: gender-transformative Aim/By-product**
				**Men only**	**Community level**						
**Jennings *et al.* 2017** Inconclusive / Mixed	Davis and Liddell	2002	Structured Videos and Discussion	Yes	No	6,7	Midwestern USA	Unknown	87	87 Male	NR
	Foshee	1998	Safe Dates:	No	Yes	6,7	North Carolina, USA	RCT	1866	48.9% male/51.1% female	NR
	Foshee	2000	Safe Dates:	No	Yes	6,7	North Carolina, USA	RCT	1866	48.9% male/51.1% female	NR
	Foshee	2005	Safe Dates	No	Yes	6,7	North Carolina, USA	RCT	1866	48.9% male/51.1% female	NR
	Schwartz *et al.*	2004	Psycho-Educational	No	No	6,7	Southern USA	RCT	58	NR	NR
	Pinzone-Glover *et al.*	1998	Discussion and Worksheets	No	No	6,7	Midwestern USA	RCT	152	59 Male/93 Female	NR
	Salazar and Cook	2006	Videos, Discussions, attendance of batterer's programme	No	No	6,7	Georgia, USA	RCT	47	47 Male	NR
**Jewell & Wormith 2010** Inconclusive / Mixed	Babcock & Steiner	1999	NR	No	No	7	NR	Quasi	355	91.8% Male	NR
	Bowen & Gilchrist	2006	NR	Yes	No	7	NR	Quasi	120	100% Male	NR
	Chang & Saunders	2002	NR	Yes	No	7	NR	Quasi	178	100% male	NR
	Dalton	2001	NR	No	No	7	NR	Quasi	85	32% Male	NR
	DeMaris	1989	NR	Yes	No	7	NR	Quasi	295	100% Male	NR
	Duplantis, Romands, & Bear	2006	NR	Yes	No	7	NR	Quasi	313	100% Male	NR
	Gerlock	2001	NR	Yes	No	7	NR	Quasi	61	100% Male	NR
	Gordon & Moriarty	2003	NR	Yes	No	7	NR	Quasi	132	100% Male	NR
**Keleher & Franklin 2008** Inconclusive / Mixed	NR	NR	Program H	NR	NR	7	Brazil	Quasi	NR	NR	Aim
	NR	NR	Program H	NR	NR	7	India	Quasi	NR	NR	Aim
	Barker *et al.*	2007	Stepping Stones	NR	NR	7	South Africa	RCT	NR	NR	Aim
**Kraft *et al.* 2014**	Daniel *et al.*	2008	Group meetings, workshops, infotainment, couples counselling (if married)	No	No	1,6,7	India	NR	NR	NR	Can't tell

**Review author(s) Conclusion:** Positive Effect No Effect Inconclusive / Mixed	**Intervention author(s)**	**Year**	**Programme name/description**	**Delivery***		**WHO Domain (1-7) †**	**Country**	**Design**	**No of Participants**	**Participants m/f**	**Intervention objective: gender-transformative Aim/By-product**
				**Men only**	**Community level**						
Inconclusive / Mixed	Daniel & Nanda	2012	See Daniel et al (2008)	No	No	1,6,7	India	NR	NR	NR	Aim
	Sebastian *et al.*	2012	"First Time Parent Project"	No	No	1,6	India	NR	NR	NR	Can't tell
	Santhya *et al.*	2008	"First Time Parent Project"	No	No	1,2,6	India	NR	NR	NR	Aim
	Shattuck *et al.*	2011	Individual education, counseling, and learning activities	Yes	No	1	Malawi	NR	NR	NR	Can't tell
	Exner *et al.*	2009	Intensive workshops (education, adult learning activities)	Yes	No	1,4	Nigeria	NR	NR	NR	Can't tell
	Schuler *et al.*	2012	Group sessions for men, women, and couples	No	No	Can't tell	Tanzania	NR	NR	NR	Aim
	Schuler & Ramirez	2012	Group sessions for men, women, and couples	No	No	Can't tell	Guatemala	NR	NR	NR	Aim
	Lundgren *et al.*	2005	Family planning education included in safe water activities, 2 in-home visits	No	No	1	El Salvador	NR	NR	NR	Can't tell
	Blake & Babalola	2002	Radio, print, community mobilisation	No	Yes	1	Guinea	NR	NR	100% male	Aim
	Kim & Marangwanda	1997	Radio, print, community mobilisation	No	Yes	1	Zimbabwe	NR	NR	100% male	Aim
	Phillips *et al.*	2012	"Navrongo Community Health and Family Planning Project"	No	Yes	1,2	Ghana	NR	NR	100% male	Aim
	Pence *et al.*	2007	Navrongo Community Health and Family Planning Project	No	Yes	1,2	Ghana	NR	NR	100% male	Aim
**Lopez *et al.* 2009** Inconclusive / Mixed	Wight *et al.*	2002	Reduce unsafe sex behaviour and unwanted pregnancy: students, 13 to 15 years	No	No	1,4,6	Scotland	RCT	8430	NR	Aim
**McCloskey *et al.* 2016**	Pulerwitz *et al.*	2010	Male Normals Initiative (MNI)	Yes	Yes	7	Ethiopia	Quasi	729	729 Male	Aim

Ruane-McAteer E, *et al. BMJ Global Health* 2019; 4:e001634. doi: 10.1136/bmjgh-2019-001634

**Review author(s) Conclusion:** Positive Effect No Effect Inconclusive / Mixed	**Intervention author(s)**	**Year**	**Programme name/description**	**Delivery***		**WHO Domain (1-7) †**	**Country**	**Design**	**No of Participants**	**Participants m/f**	**Intervention objective: gender-transformative Aim/By-product**
				**Men only**	**Community level**						
Inconclusive / Mixed	Wagman *et al.*	2015	SHARE	No	Yes	4, 7	Uganda	RCT	11448	NR	Aim
	Ambramsky *et al.*	2014	SASA!	No	Yes	4,7	Uganda	RCT	2776	1360 Males/1416 Females	Aim
	Jewkes *et al.*	2008	Stepping Stones	No	No	4,6	South Africa	RCT	2776	1360 Males/1416 Females	Aim
	Proynk *et al.*	2008	IMAGE	No	No	4,7	South Africa	RCT	23376	NR	Aim
	Keller *et al.*	2015	Your Moment of Truth (YMOT)	Yes	No	6,7	Kenya	Quasi	1836	1836 Male	Aim
**Napierala Mavedzenge *et al.* 2011 *interventions grouped by programme rather than by study*** Inconclusive / Mixed	Ross *et al.*	2007	MEMA kwa Vijana	No	No	1,4,6	Tanzania	RCT	9645	55% Male/ 45% Female	Aim
	Terris-Prestholt *et al.*	2006	MEMA kwa Vijana	No	No	1,4,6	Tanzania	RCT	9645	55% Male/ 45% Female	Aim
	Hayes *et al.*	2005	MEMA kwa Vijana	No	No	1,4,6	Tanzania	RCT	9645	55% Male/ 45% Female	Aim
	Osabi *et al.*	2006	MEMA kwa Vijana	No	No	1,4,6	Tanzania	RCT	9645	55% Male/ 45% Female	Aim
	Doyle *et al.*	2009	MEMA kwa Vijana	No	No	1,4,6	Tanzania	RCT	9645	55% Male/ 45% Female	Aim
	James *et al.*	2006	Department of Education Life Skills Program	No	No	4,6	South Africa	Quasi	1141	49% Male/ 51% Female	Aim
	Cowan *et al.*	2009	Regai Dzive Shiri	No	No	1,4,6,	Zimbabwe	RCT	6791	52% Male/ 48% Female	Aim
	Hayes *et al.*	2005	Regai Dzive Shiri	No	No	1,4,6	Zimbabwe	RCT	6791	52% Male/ 48% Female	
	Jewkes *et al.*	2007	Stepping Stones	NR	No	4,6	South Africa	NR	2776	NR	NR
	Jewkes *et al.*	2006	Stepping Stones	NR	No	4,6	South Africa	RCT	2776	NR	
	Jewkes *et al.*	2008	Stepping Stones	NR	No	4,6	South Africa	RCT	2776	NR	
	Hargreaves	2009	IMAGE	No	No	1,4,6	South Africa	RCT	3895	56% Female/ 44% Male	Aim
	Proynk *et al.*	2008	IMAGE	No	No	1,4,6	South Africa	RCT	3895	56% Female/ 44% Male	
	Proynk *et al.*	2008	IMAGE	No	No	1,4,6	South Africa	RCT	3895	56% Female/ 44% Male	
**Remme *et al.* 2014**	Jewkes *et al.*	2008	Stepping Stones	No	Yes	4,6,7	South Africa	RCT	70 study clusters comprised 64 villages and 6 townships	1360 men; 1416 women	Aim

Review author(s) Conclusion: Positive Effect No Effect Inconclusive / Mixed	Intervention author(s)	Year	Programme name/description	Delivery*		WHO Domain (1-7) †	Country	Design	No of Participants	Participants m/f	Intervention objective: gender-transformative Aim/By-product
				Men only	Community level						
Inconclusive / Mixed	Pulerwitz *et al.*	2006	Intervention (a) interactive group education sessions for young men led by adult male facilitators. Intervention (b) group education community-wide ''lifestyle'' social marketing campaign to promote condom use using gender-equitable messages.	Yes	Yes	4,6,7	Brazil	Quasi	2 sites1 control site 508 young men	100% men	Aim
	Verma *et al.*	2006	See Pulerwitz et al (2006) above	Yes	Yes	4,6,7	India	Quasi	1423 married and unmarried young men aged 1629 in urban settings and aged 15 24 in rural settings	100	Aim
	Abramsky *et al.*	2014	SASA!	No	Yes	4,6,7	Uganda	RCT	4 intervention and 4 control communities Random sample of adult community members sampled at baseline (n1583) and postintervention (n2532)	NR	Aim
**Ricardo *et al.* 2011* Only moderate and high quality study details reported**	Foshee *et al.*	1998; 2000; 2004	NR	No	NR	1,7	USA	Unclear if RCT or Quasi	n=460	41.5% male	Aim
	Gidycz *et al.*	2011	NR	Yes	NR	1,7	USA	Unclear if RCT or Quasi	n=494	100% male	Aim
	Jewkes *et al.*	2008	NR	No	NR	1,7	South Africa	Unclear if RCT or Quasi	n=2776	49% male	Aim
Positive Effect	Taylor *et al.*	2010	NR	No	NR	1,7	USA	Unclear if RCT or Quasi	n=1592	48% male	Aim

Ruane-McAteer E, *et al. BMJ Global Health* 2019; 4:e001634. doi: 10.1136/bmjgh-2019-001634

**Review author(s) Conclusion:** Positive Effect No Effect Inconclusive / Mixed	**Intervention author(s)**	**Year**	**Programme name/description**	**Delivery***		**WHO Domain (1-7) †**	**Country**	**Design**	**No of Participants**	**Participants m/f**	**Intervention objective: gender-transformative Aim/By-product**
				**Men only**	**Community level**						
	Gidycz *et al.*	2001	NR	No	NR	1,7	USA	Unclear if RCT or Quasi	n=1108	27% male	Aim
	Lobo	2004	NR	Yes	NR	1,7	USA	Unclear if RCT or Quasi	n=237	100% male	Aim
	Kantor	No date	NR	No	NR	1,7	USA	Unclear if RCT or Quasi	n=157	42% male	Aim
	Stephens & George	2009	NR	Yes	NR	1,7	USA	Unclear if RCT or Quasi	n=65	100% male	Aim
	Jaycox *et al.*	2006	NR	No	NR	1,7	USA	Unclear if RCT or Quasi	n=2540	48% male	Aim
	Kerpelman *et al.*	2009	NR	NR	NR	1,7	USA	Unclear if RCT or Quasi	n=249	NR	Aim
	Wolfe *et al.*	2009	NR	No	NR	1,7	Canada	Unclear if RCT or Quasi	n=1722	47.2% male	Aim
	Gardner & Boellaard	2007	NR	No	NR	1,7	USA	Unclear if RCT or Quasi	n=150	19.4% male	Aim
	Pulerwitz *et al.*	2010	NR	Yes	NR	1,7	Ethiopia	Unclear if RCT or Quasi	n=645	100% male	Aim
	Verma *et al.*	2008	NR	Yes	NR	1,7	India	Unclear if RCT or Quasi	n=1137	100% male	Aim
	Wolfe *et al.*	2003	NR	No	NR	1,7	Canada	Unclear if RCT or Quasi	n=158	50% male	Aim
	Banyard *et al.*	2007	NR	No	NR	1,7	USA	Unclear if RCT or Quasi	n=389	44% male	Aim
	Davis & Liddell	2002	NR	Yes	NR	1,7	USA	Unclear if RCT or Quasi	n=87	100% male	Aim

**Review author(s) Conclusion:** Positive Effect No Effect Inconclusive / Mixed	**Intervention author(s)**	**Year**	**Programme name/description**	**Delivery***		**WHO Domain (1-7) †**	**Country**	**Design**	**No of Participants**	**Participants m/f**	**Intervention objective: gender-transformative Aim/By-product**
				**Men only**	**Community level**						
	Fay & Medway	2006	NR	No	NR	1,7	USA	Unclear if RCT or Quasi	n=154	44% male	Aim
	Foubert & Marriot	1997	NR	Yes	NR	1,7	USA	Unclear if RCT or Quasi	n=77	100% male	Aim
	Hillenbrand-Gunn *et al.*	2010	NR	No	NR	1,7	USA	Unclear if RCT or Quasi	n=212	60% male	Aim
	Macgowan	1997	NR	No	NR	1,7	USA	Unclear if RCT or Quasi	n=440	43.9% male	Aim
	Roberts	2009	NR	No	NR	1,7	USA	Unclear if RCT or Quasi	n=332	49% male	Aim
	Soul City	2006	NR	No	NR	1,7	South Africa	Unclear if RCT or Quasi	n=1877	49% male	Aim
	Weisz & Black	2001	NR	No	NR	1,7	USA	Unclear if RCT or Quasi	n=66	42% male	Aim
	Gruchow & Brown	2011	NR	Yes	NR	1,7	USA	Unclear if RCT or Quasi	n=230	100% male	Aim
	Pulerwitz *et al.*	2006	NR	Yes	NR	1,7	Brazil	Unclear if RCT or Quasi	n=609	100% male	Aim
	Solórzano *et al.*	2008	NR	No	NR	1,7	Nicaragua	Unclear if RCT or Quasi	n=4800	46% male	Aim
	Winkel & DeKleuver	1997	NR	No	NR	1,7	Netherlands	Unclear if RCT or Quasi	n=198	31% male	Aim
	Moynihan *et al.*	2011	NR	No	NR	1,7	USA	Unclear if RCT or Quasi	n=98	56.8% male	Aim
	Moynihan *et al.*	2010	NR	No	NR	1,7	USA	Unclear if RCT or Quasi	n=98	56.8% male	Aim

Review author(s) Conclusion: Positive Effect No Effect Inconclusive / Mixed	Intervention author(s)	Year	Programme name/description	Delivery*		WHO Domain (1-7) †	Country	Design	No of Participants	Participants m/f	Intervention objective: gender-transformative Aim/By-product
				Men only	Community level						
**Sarkar et al 2015** Positive Effect	Shattuck *et al.*	2011	MMM (Malawi Male Motivator)	No	No	1	Malawi	RCT	NR	NR	Aim
**Skevington et al 2013** Positive Effect	Pacific Regional HIV/AIDS Project	2007	Stepping Stones	No	Yes	4,6	Fiji	RCT	627	NR	Aim
	Hadjipateras *et al.*	2006	Stepping Stones	No	Yes	4,6	Angola	RCT	633	NR	Aim
	Hadjipateras *et al.*	2006	Stepping Stones	No	Yes	4,6	Tanzania	RCT	674	NR	Aim
	Hadjipateras *et al.*	2006	Stepping Stones	No	Yes	4,6	Uganda	RCT	1478	NR	Aim
	Bradley *et al.*	2009	Stepping Stones	No	Yes	4,6	India	RCT	6800	NR	Aim
	Jewkes *et al.*	2006	Stepping Stones	Yes	Yes	4,6	South Africa	RCT	2776	1360 Men/1416 Women	Aim
	Pain *et al.*	2002	Stepping Stones	No	Yes	4,6	The Gambia	RCT	945	NR	Aim
	Bhattacharjee and Costigan	2005	Stepping Stones	No	Yes	4,6	Ethiopia	RCT	696	NR	Aim
	Jurjue *et al.*	2000	Stepping Stones	No	Yes	4,6	The Gambia	RCT	NR	NR	Aim
**Small *et al.* 2013** Positive Effect	Paine *et al.*	2002	Stepping stones:	No	Yes	4	Gambia	Quasi	563	NR	Aim
	Jewkes *et al.*	2008	Stepping stones:	No	Yes	4,6	South Africa	RCT	2776	1,360 men/1,416 women	Aim
	Kalichman *et al.*	2009	Integrated gender-based violence and HIV risk reduction intervention: 'gender tailored' to men, curriculum on gender inequalities	Yes	Yes	4	South Africa	Quasi	475	100% male	Aim
**Smedslund *et al.* 2007** Inconclusive / Mixed	Labriola *et al.*	2005	Bronx Experiment - CBT v No treatment	Yes	No	7	USA	RCT	420	100% male	Aim
	Davis *et al.*	2000	Brooklyn Experiment - CBT v No treatment	Yes	No	7	USA	RCT	376	100% male	Aim
	Maxwell *et al.*	2004	Brooklyn Experiment - CBT v No treatment	Yes	No	7	USA	RCT	376	100% male	Aim
	Taylor *et al.*	2001	Brooklyn Experiment - CBT v No treatment	Yes	No	7	USA	RCT	376	100% male	Aim

**Review author(s) Conclusion:** Positive Effect No Effect Inconclusive / Mixed	**Intervention author(s)**	**Year**	**Programme name/description**	**Delivery***		**WHO Domain (1-7) †**	**Country**	**Design**	**No of Participants**	**Participants m/f**	**Intervention objective: gender-transformative Aim/By-product**
				**Men only**	**Community level**						
	Feder & Dugan	2002	Broward Experiment - CBT v No treatment	Yes	No	7	USA	RCT	404	100% male	Aim
	Feder & Forde	2000	Broward Experiment - CBT v No treatment	Yes	No	7	USA	RCT	404	100% male	Aim
	Dunford	2000	San Diego Navy - CBT v No treatment	No	No	7	USA	RCT	861 couples - randomly assigned to four groups: a men's group (N = 218); a conjoint group (the men participating with their wives) (N = 216); a rigorously monitored group (N = 213), and a control group (N = 214) who received no treatment.	50% male (?)	Aim
	Dunford	2000	San Diego Navy - CBT v No treatment	No	No	7	USA	RCT	861 couples - randomly assigned to four groups: a men's group (N = 218); a conjoint group (the men participating with their wives) (N = 216); a rigorously monitored group (N = 213), and a control group (N = 214) who received no treatment.	50% male (?)	Aim
	Saunders	1996	Wisconsin Study - CBT v Other therapy	Yes*	No	7	USA	RCT	217	100% male* (The partners of 79% of the 136 treatment completers gave reports of the men's behaviour an average of 2 years post-treatment.)	Aim
	Easton	2005	Yale Study - CBT v Other therapy	Yes*	No	7	USA	RCT	64	100% male* (collateral reports from female partners at baseline, monthly, and post-treatment periods)	Aim

**Review author(s) Conclusion:** Positive Effect No Effect Inconclusive / Mixed	**Intervention author(s)**	**Year**	**Programme name/description**	**Delivery***		**WHO Domain (1-7) †**	**Country**	**Design**	**No of Participants**	**Participants m/f**	**Intervention objective: gender-transformative Aim/By-product**
				**Men only**	**Community level**						
	Easton *et al.*	2007	Yale Study - CBT v Other therapy	Yes*	No	7	USA	RCT	64	100% male* (collateral reports from female partners at baseline, monthly, and post-treatment periods)	Aim
	Easton *et al.*	2007	Yale Study - CBT v Other therapy	Yes*	No	7	USA	RCT	64	100% male* (collateral reports from female partners at baseline, monthly, and post-treatment periods)	Aim
**Storer *et al.* 2016** Positive Effect	Gidycz *et al.*	2011	"The Men's Project"	No	No	7	USA	RCT	635	NR	Aim
	Miller *et al.*	2012	"Coaching Boys into Men"	Yes	Yes	6,7	USA	Quasi	847 (intervention); 951 (control)	100% male	Aim
**Tokhi *et al.* 2018** Inconclusive / Mixed	Kunene *et al.*	2005	Facility-based couples' education at antenatal clinics	No	No	2	South Africa	RCT	Intervention: 1337; Control: 1252	Men: 45.47% (intervention), 44.57% (control)	By-product
	Midhet *et al.*	2010	Community-based education for men and women; training of traditional birth attendants; and community mobilisation to improve referral	No	Yes	2	Pakistan	RCT	Intervention: 703; Comparison: 838; Control: 1022	NR	By-product
	Mullany *et al.*	2007	Facility-based couples' education at antenatal clinics at a tertiary hospital	No	No	2	Nepal	RCT	Intervention: 145; Comparison: 148; Control: 149	NR	By-product
	Sahip *et al.*	2007	Workplace-based education for expectant fathers	Yes	No	2	Turkey	Quasi	Intervention: 80; Control: 80	NR	By-product
	Varkey *et al.*	2004	Facility-based education for men and women individually or as a couple at antenatal clinics	No	No	2	India	Quasi	Intervention:654; Control: 604	50% male	By-product

Ruane-McAteer E, *et al. BMJ Global Health* 2019; 4:e001634. doi: 10.1136/bmjgh-2019-001634

**Review author(s) Conclusion:** Positive Effect No Effect Inconclusive / Mixed	**Intervention author(s)**	**Year**	**Programme name/description**	**Delivery***		**WHO Domain (1-7) †**	**Country**	**Design**	**No of Participants**	**Participants m/f**	**Intervention objective: gender-transformative Aim/By-product**
				**Men only**	**Community level**						
	Fullerton *et al.*	2005	Family and community education (home visits and group discussions)	No	Yes	2	India	Quasi	Baseline: 600; Post intervention: 833	NR	By-product
	Hossain *et al.*	2006	Family and community education (home visits and group discussions) and improved community-facility linkages	No	Yes	2	Bangladesh	Quasi	NR	NR	By-product
**Arango *et al.* 2014** Inconclusive / Mixed	Individual intervention study detail not reported										
**Bakrania *et al.* 2018** Inconclusive / Mixed	Individual intervention study detail not reported										
**Chatterjee 2015** Inconclusive / Mixed	Individual intervention study detail not reported										
**Hartmann *et al.* 2016** Inconclusive / Mixed	Individual intervention study detail not reported										
**Krishnaratne *et al.* 2016** Inconclusive / Mixed	Individual intervention study detail not reported										
**Lacroix *et al.* 2013** Positive Effect	Individual intervention study detail not reported										
**Muralidharan *et al.* 2014**	Individual intervention study detail not reported										

Ruane-McAteer E, *et al. BMJ Global Health* 2019; 4:e001634. doi: 10.1136/bmjgh-2019-001634

**Review author(s) Conclusion:** Positive Effect No Effect Inconclusive / Mixed	**Intervention author(s)**	**Year**	**Programme name/description**	**Delivery***		**WHO Domain (1-7) †**	**Country**	**Design**	**No of Participants**	**Participants m/f**	**Intervention objective: gender-transformative Aim/By-product**
				**Men only**	**Community level**						
Positive Effect											
**Rankin *et al.* 2016** Inconclusive / Mixed	Individual intervention study detail not reported										
**Rees *et al.* 2014** Inconclusive / Mixed	Individual intervention study detail not reported										
**Schriver *et al.* 2017** Inconclusive / Mixed	Individual intervention study detail not reported										

*Not reported (NR)
†WHO Domain (1-7): 1. Helping people realize their desired family size; 2. Ensuring the health of pregnant women and girls and their new-born infants; 3. Preventing unsafe abortion; 4.Promoting sexual health and well-being; 5. Promoting sexual and reproductive health in disease outbreaks; 6. Promoting healthy adolescence for a healthy future, and unsafe abortion; harmful traditional practices, child, early, and forced marriage; and sexual coercion and intimate partner violence; 7. Preventing and responding to violence against women and girls and harmful practices.
